# Enhancing Biomethane Production from Corn Stover: Insights into Lignocellulosic Component Interactions and Pretreatment Efficacy

**DOI:** 10.3390/bioengineering13060630

**Published:** 2026-05-28

**Authors:** Xiteng Chen, Lu Liu, Hairong Yuan, Xiujin Li

**Affiliations:** Department of Environmental Science and Engineering, Beijing University of Chemical Technology, Beijing 100029, China; 2020400061@buct.edu.cn (X.C.);

**Keywords:** anaerobic digestion, biomethane, lignocellulosic individual components, synthetic mixtures, corn stover, pretreatment

## Abstract

In this study, the methane yield, substance conversion rate and microbial community structure of individual components of lignocellulose, synthetic mixtures, and corn straw subjected to different pretreatments (thermal hydrolysis, chemical, biological, and combined pretreatment) during anaerobic digestion were comparatively investigated. The synthetic mixture of cellulose and hemicellulose (MCXY) exerted a positive promoting effect on biomethane production, with a synergistic effect index of 101.51%. The methane yield per volatile solids (VS) of microcrystalline cellulose (MC), xylan (XY), and MCXY reached 320.81 ± 1.85 mL/g VS, 352.70 ± 6.58 mL/g VS, and 340.60 ± 10.94 mL/g VS, respectively. Lignin did not produce biogas in anaerobic digestion (AD) system, and its presence had an inhibitory effect on the methanogenesis of cellulose and hemicellulose, especially that of hemicellulose. Notably, pretreatment significantly improved the methane production potential of corn stover. Deep eutectic solvent-pretreated corn stover (DES_CS) achieved the highest methane yield of 356.57 ± 8.50 mL/g VS, which was 55.46% higher than that of the untreated group. DES pretreatment deconstructed lignocellulosic matrix and distinctly increased DOM molecular diversity, thus providing superior substrate conditions for improving anaerobic digestion performance. Microbial community analysis revealed that DES pretreatment significantly reshaped the bacterial structure, enriching syntrophic taxa over the carbohydrate-degrading *Bacteroides* found in raw corn stover, thereby fostering a more robust metabolic network for methane production. While acetoclastic *Methanothrix* dominated the pretreated groups, its synergistic coexistence with hydrogenotrophic *Methanobacterium* across all digesters facilitated stable dual-pathway methanogenesis. This work can provide a theoretical basis and technical reference for the optimization and application of pretreatment strategies for efficient anaerobic digestion of corn stover.

## 1. Introduction

As an eco-friendly biotechnology, anaerobic digestion (AD) technology facilitates the reduction, resource recycling, and energy utilization of biomass waste. China produces enormous quantities of agricultural biomass waste every year, as a major agricultural nation [1]. Based on publicly available data from the China Rural Statistical Yearbook (2001–2024), combined with the grain-to-straw ratio and effective collection coefficients of straw for various provinces across China [2], the national potential output of crop straw increased from 552 million tons in 2000 to 857 million tons in 2024. AD offers a promising approach to transform environmental concerns into renewable energy resources [3,4]. As the primary product of anaerobic digestion plants, biogas mainly consists of methane (50–70%) and carbon dioxide (30–50%), and thus serves as a valuable renewable energy carrier [5]. It can be directly applied for heating and power generation or upgraded to inject into natural gas grids.

With its abundance and cost-effectiveness, corn stover is an ideal feedstock for biomethane production [6]. However, its recalcitrant lignocellulosic structure—specifically the tight cross-linking and lignin-mediated encapsulation of polysaccharides—restricts the access of enzymes and microorganisms to biodegradable components [7,8,9]. In addition, synergistic or antagonistic interactions among structural components further interfere with hydrolysis, acidification, and methanogenesis [10]. Hydrolysis is widely regarded as the rate-limiting step in the anaerobic digestion of lignocellulosic biomass [11,12]. Consequently, maximizing the methane yield from corn stover by overcoming its structural recalcitrance is a priority for sustainable biomass utilization [13]. Numerous studies have employed various pretreatment methods, including mechanical pretreatment, hydrothermal treatment, acid–alkali chemical treatment, biological pretreatment, enzyme pretreatment, and combined methods to disrupt the straw structure, and improve substrate accessibility and anaerobic digestion efficiency [14,15,16,17]. Bai et al. confirmed that KOH pretreatment enhanced the bioaccessibility and methane recovery of lignocellulosic substrates derived from dewatered paunch solids, achieving a maximum promotion rate of 150% [15]. Sha et al. investigated the influence of alkaline deep eutectic solvent (DES) pretreatment on methane production during anaerobic digestion of corn stover, and reported that the maximum methane yield was enhanced by 1.16 times [18]. Lu et al. used a low-temperature urea-hydrothermal method to treat corn stover, which increased biomethane yield by 19.1% and shortened digestion time by three days [19]. Despite considerable progress made in relevant pretreatment research, several limitations persist. Firstly, most existing studies focus on the effects of pretreatment on the overall structural disruption of straw and the enhancement of biogas production performance, while few studies have provided a comparative analysis of methanogenic performance from the perspective of individual lignocellulosic components and their complex interactions. Secondly, although some studies have investigated the anaerobic biogas production patterns of single-component or mixed-component lignocellulosic systems, these findings have not been effectively correlated with the actual pretreatment systems of raw corn stover [10,20]. This makes it challenging for the experimental results obtained from monomer-component systems to directly guide the optimization of high-efficiency pretreatment processes for natural straw. Studies combining emerging DES pretreatment with anaerobic digestion comparison of individual lignocellulosic components also remain relatively scarce. Furthermore, systematic comparative analyses of the microbial community composition among individual lignocellulosic components, synthetic mixtures, and raw corn stover remain limited.

This study aims to systematically investigate the methanogenic performance and microbial community characteristics of lignocellulosic biomass through a multi-tiered comparative approach. Specifically, the objectives are to: (1) systematically evaluate the methanogenic performance and biomethane potential of individual components (cellulose, hemicellulose, and lignin), their synthetic mixtures, and corn stover (both raw and pretreated); (2) identify optimal pretreatment strategies by performing a longitudinal comparison of substrate conversion rates between individual components and pretreated corn stover; (3) compare the microbial community structures across diverse anaerobic digestion systems to characterize the differences between simplified component systems and complex biomass systems; (4) elucidate the methanogenic metabolic pathways within anaerobic digestion systems specifically characterized by lignin removal or the separation of cellulose and hemicellulose. These findings are expected to provide a theoretical foundation and practical guidance for optimizing high-efficiency pretreatment strategies for corn stover, elucidating the synergistic degradation mechanism of lignocellulose, and maximizing methane production capacity.

## 2. Materials and Methods

### 2.1. Substrate Materials and Inoculum

The individual components of lignocellulose include lignin, cellulose, and hemicellulose. Alkaline lignin (S19288, Shanghai Yuanye Bio-Technology Co., Ltd., Shanghai, China), microcrystalline cellulose (M909921, Shanghai Macklin Biochemical Co., Ltd., Shanghai, China), and xylan from corncob (X820567, Shanghai Macklin, Shanghai, China) were used as model compounds for lignin, cellulose, and hemicellulose, respectively, as shown in Table 1. Corn stover was collected from a farm in Yanqing District, Beijing. After natural air-drying, impurities such as gravel and soil clods were removed, and corn stover was crushed to 20 mesh using a crusher. Anaerobic granular sludge exhibited outstanding organic matter degradation performance and remarkable anaerobic methanogenesis potential in our previous research. Anaerobic granular sludge (AGS), which possessed excellent organic matter degradation performance and anaerobic methane production potential, was used as inoculum in this study [21]. The inoculum was used for anaerobic digestion experiments only after degassing in a water bath at 35 °C [22]. Table 1 lists the main characteristics of the substrates and inoculum.

### 2.2. Experimental Design

#### 2.2.1. Pretreatment

The mixing ratio of synthetic mixtures was determined by measuring the actual contents of cellulose, hemicellulose, and lignin in corn stover. Microcrystalline cellulose (MC), xylan (XY) and alkaline lignin (LI) were mixed at a ratio of 7:6:1 (cellulose: hemicellulose: lignin, based on TS (total solids)). The synthetic mixtures, (binary mixture of cellulose and lignin (MCLI), binary mixture of hemicellulose (xylan) and lignin (XYLI), binary mixture of cellulose and hemicellulose (xylan) (MCXY), ternary mixture of cellulose, hemicellulose (xylan) and lignin (MXL)), are shown in Table 2. Prior to mixing, cellulose, hemicellulose, and lignin were dried to eliminate residual moisture and sieved with a designated mesh screen. Each component was accurately weighed based on preset mass ratios, placed into an agate mortar, and ground homogenously for 10 min.

Table 3 showed different pretreatment of corn stover: untreated CS, thermally hydrolysis pretreated CS (H-CS), thermally hydrolysis-urea co-pretreated CS (HU-CS), biogas slurry-pretreated CS (BS-CS), CaO-pretreated CS (CaO-CS), biogas slurry-CaO co-pretreated CS (BC-CS), and deep eutectic solvent-pretreated CS (DES-CS). The biogas slurry was collected from the effluent of a food waste treatment plant in Beijing, China. The TS, VS, and pH were 2.1%, 0.9%, and 7.3, respectively. After DES pretreatment, the solid residues were washed thoroughly with ethanol–water mixed solution (volume ratio = 4:1) until the washing filtrate became neutral, and then dried in an oven. The lignocellulosic components of different pretreatment groups were determined, as shown in the Table 4.

#### 2.2.2. Batch Anaerobic Digestion

Batch anaerobic digestion of individual lignocellulosic components, synthetic mixtures, and various pretreated samples was performed using an AMPTS II system (BPC Instruments, China), with anaerobic granular sludge serving as the inoculum. Mesophilic anaerobic digestion (35 ± 0.5 °C) was performed with a reaction volume of 400 mL. The carbon-to-nitrogen ratio and initial pH were adjusted to 25:1 and 7.5, respectively, before the formal operation of the AD reactors. The inoculation ratio was set as 2:1 (AGS: Substrate, calculated on volatile solids (VS)), the organic loading rate was set as 20 g VS/L, and total solids (TS) content of the fermentation system was controlled at approximately 8%. Triplicate replicates were set for each experimental group, and a separate control group with only inoculum sludge was established to subtract the background biogas production from the inoculum. During the 25-day AD period, daily biogas yields and compositions were recorded and subsequently normalized to standard conditions (273.15 K, 101.325 kPa). Daily methane production was determined by multiplying the daily biogas volume by its corresponding methane proportion.

### 2.3. Analytical Methods

#### 2.3.1. Physical and Chemical Properties

Biogas composition was analyzed using a gas chromatograph (GC-2014c, Shimadzu, Kyoto, Japan). The instrument was equipped with a TDX-01 chromatographic column and a thermal conductivity detector (TCD), employing high-purity argon as the carrier gas at a constant flow rate of 30 mL/min. TS, VS, and pH value were determined using the standard method issued by APHA (2012) [23]. The contents of three components of lignocellulose (lignin, cellulose, and hemicellulose) were analyzed with a fully automatic cellulose analyzer (Model F2000, Hanon Instruments, Jinan, China). Prior to lignocellulose content determination, all test samples were obtained via uniform sampling, dried at 105 °C, then crushed, ground, and sieved through a 10-mesh screen. The mass of sample loaded into each filter bag ranges from 0.5 g to 1 g. Ethanol and volatile fatty acids (VFAs) were analyzed using gas chromatography with high-purity nitrogen as the carrier gas (GC-2014c, Shimadzu, Japan). Fourier transform ion cyclotron resonance mass spectrometry (FT-ICR-MS) analyses for dissolved organic matter (DOM) were performed using a SoleriX 15T FT-ICR-MS platform (Bruker Daltonics GmbH & Co. KG, Bremen, Germany), employing an electrospray ionization (ESI) source in negative ion mode [21]. The raw water sample (12 mL) was filtered through a 0.22 μm filter to eliminate particulates and impurities, then acidified by dropwise addition of formic acid to adjust the sample pH to 2. Solid-phase extraction of DOM in the sample was subsequently carried out with an Agilent Bond Elut PPL cartridge (1.0 g, 6 mL). Prior to sample detection, the instrument was calibrated with a 10 mmol/L sodium format solution. After sample analysis, internal standard calibration was performed using soluble organic matter with a known molecular formula, and all mass errors of the detections were less than 1 ppm following calibration. Calculations related to DOM components were conducted on the online platform (https://domnetweb.com) for subsequent statistical analysis.

#### 2.3.2. Kinetic Equations

The Modified Gompertz and Cone model equations were used to fit the cumulative biomethane yield, as shown in Equations (1) and (2). In the formulas, the cumulative methane production (mL/g VS) was represented by *B*. The maximum potential methane production (mL/g VS) was referred to as *B*_0_, and the maximum methane production rate (mL/g VS/d) was represented by *R*_max_. *λ* was the lag phase (d), *t* was the reaction time (d), and *k* was the rate constant (d^−1^). The symbol e was defined as a mathematical constant with an approximate value of 2.7183. *n* was the shape factor, a dimensionless parameter that describes the shape of the methane production curve.

(1) $$B\;=\;B_{0}\;\times \;\exp\left\{-\exp\left[\frac{R_{\max}\;\times \;e}{B_{0}}\left(\lambda -t\right)+1\right]\right\}\;$$

(2) $$B\;=\;\frac{B_{0}}{1+{\;\left(k\;\times \;t\right)}^{-n}}$$

#### 2.3.3. Synergistic Effect Index

The synergistic effect index (SEI) was adopted to evaluate the interaction among the three major components of lignocellulose in terms of methane production, and its calculation formula was presented as follows (Equations (3) and (4)). EMP (experimental cumulative methane production) was used to denote the experimental methane production of different experimental groups, while CMP (calculated theoretical cumulative methane production) was referred to as the calculated value of theoretical cumulative methane yield. This value was obtained by summing the methane production from the individual fermentation of lignin, cellulose, and hemicellulose. *P*_i_ was defined as the proportion of each component in the synthetic mixtures.

(3) $$\mathrm{Synergistic}\;\mathrm{index}\;(\mathrm{SEI})=\;\frac{\mathrm{EMP}}{\mathrm{CMP}}\;\times \;100\%$$

(4) $$\mathrm{CMP}\;=\;P_{\mathrm{MC}}\;\times \;{\mathrm{EMP}}_{\mathrm{MC}\;}+{\;P}_{\mathrm{XY}}\;\times {\;\mathrm{EMP}}_{\mathrm{XY}}\;+{\;P}_{\mathrm{LI}}\;\times \;{\mathrm{EMP}}_{\mathrm{LI}\;}$$

### 2.4. 16S rRNA Gene Amplicon Sequencing and Metagenomic Analysis

Samples collected from anaerobic digestion reactors were preserved at −80 °C before subsequent processing. Total microbial genomic DNA was extracted from samples using E.Z.N.A.^®^ Soil DNA Kit (Omega Bio-tek, Norcross, GA, USA) following standard protocols. Microbial community composition was characterized via 16S rRNA gene amplicon sequencing on Illumina NextSeq 2000 platform (Illumina Inc., San Diego, CA, USA). The V3-V4 hypervariable region of bacterial 16S rRNA gene was amplified with primers 338F (5′-ACTCCTACGGGAGGCAGCAG-3′) and 806R (5′-GGACTACHVGGGTWTCTAAT-3′). Archaeal sequences were amplified using primer pairs 524F10ext (5′-TGYCAGCCGCCGCGGTAA-3′) and Arch958Rmod (5′-YCCGGCGTTGAVTCCAATT-3′).

Whole metagenomic sequencing was conducted on Illumina NovaSeq™ X Plus platform (Illumina Inc., San Diego, CA, USA). Raw data were processed on Majorbio Cloud Platform (www.majorbio.com, assessed on 10 April 2026) [24]. Low-quality sequences, chimeras, and contaminant reads were removed. Valid clean reads were assembled with MEGAHIT v1.1.2. Gene sequences were taxonomically and functionally annotated against NCBI and KEGG databases.

### 2.5. Statistical Analysis

Microsoft Excel 2021 was used for data recording and calculation. Prior to ANOVA, the normality of cumulative methane yield data was assessed using the Shapiro–Wilk test, and homogeneity of variance was evaluated using Levene’s test. No significant violation of the assumptions for one-way ANOVA was detected. Statistical analysis of cumulative methane yield was conducted using one-way ANOVA followed by Tukey’s post hoc test in SPSS 26.0, with *p* < 0.05 considered statistically significant. Kinetic fitting and graph plotting were performed using Origin 2026. Raw functional gene abundances were subjected to Z-score normalization in R (version 4.5.3) to achieve dimensionality unification. Multiple-group heatmaps of batch matrices were generated using the CNSKnowall platform (https://www.cnsknowall.com, accessed on 2 May 2026).

## 3. Results and Discussion

### 3.1. Biomethane Production

**Figure 1:**
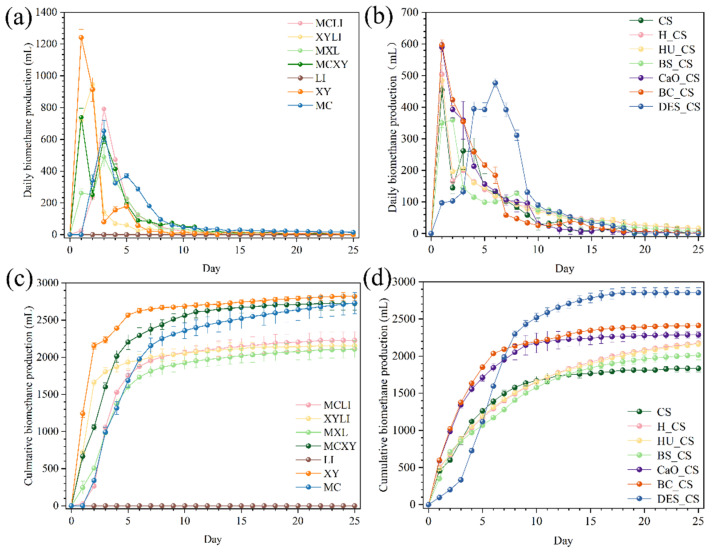
Daily biomethane production (**a**,**b**) and cumulative biomethane production (**c**,**d**) in lignocellulosic individual components and corn stover anaerobic digestion groups.

As presented in Figure 1a, the results of daily biomethane production revealed the biogas production characteristics of different lignocellulosic components, as well as their intricate interactive behaviors during co-fermentation. The first primary peak of methane production in MC group was observed on the third day (652.38 ± 66.69 mL), followed by a secondary peak which was recorded on the fifth day. Conversely, the highest daily methane production peak for the hemicellulose AD system was reached on the first day (1241.15 ± 50.08 mL). With a simpler structural composition, hemicellulose was more vulnerable to hydrolytic degradation by anaerobic microorganisms [21]. Consequently, its methanogenesis process initiated earlier than that of cellulose, and contributed to a higher methane yield. In addition, no biomethane was detected from lignin, indicating that lignin was poorly converted into biogas by anaerobic microorganisms [25,26]. Lignin could not be degraded by anaerobic microorganisms for methane production, and it exerted a negative impact on biogas production when mixed with other substances [10,27]. After lignin was mixed with cellulose and hemicellulose, respectively, the overall biomethane production trends were observed to be consistent with those of the pure individual components. However, both the cumulative methane production and the cumulative specific methane yield were significantly suppressed compared to the non-lignin addition systems (Figure 1c). Accordingly, the synergistic effect index of MCLI and XYLI were determined to be less than 100% (Table 5). The daily methane production profile of the cellulose–hemicellulose binary mixture was highly consistent with those of its individual components, exhibiting two distinct methane production peaks (738.17 ± 58.17 mL on the first day and 611.47 ± 35.75 mL on the third day). Co-fermentation of cellulose and hemicellulose (MCXY) exhibited a promoting effect on biomethane production, with a SEI of 101.5%. Although the promoting effect on biomethane production was not significant in the results of this study, previous literature had confirmed this remarkable synergistic effect on methane production [10]. Furthermore, the initial daily methane peak on the first day for MXL was substantially lower than that of the MCXY group, and the cumulative biogas methane yield was the lowest, only reaching 2110.5 ± 116.36 mL. According to the cumulative specific methane yield (CSMY) excluding lignin VS and SEI of synthetic mixtures (Table 5), lignin was found to exert a negative effect on the anaerobic methanogenesis of cellulose and hemicellulose, and a markedly stronger inhibitory effect was observed on hemicellulose.

According to CSMY, excluding lignin of XYLI and XY, the methane yield of XYLI decreased by 8.55%. In the MXL group, the biomethane production process of hemicellulose was restricted by lignin, and the methane yield of MXL was thereby influenced. Different pretreatment methods, which modify straw structure or achieve delignification, could affect the biomethane production performance of corn straw. The maximum daily methane production occurred on the first day, after which the daily methane yields gradually declined in all corn stover pretreatment groups except DES_CS (Figure 1b). For the corn stover without pretreatment group, a primary methane production peak appeared on the first day of anaerobic digestion, and a secondary peak was also observed on the fourth day. CS group had the lowest cumulative methane production among all treatments, which was 1832.64 ± 46.25 mL. The BS_CS group had the second-lowest cumulative methane production. The cumulative methane production of the combined pretreatment BC_CS group was 2411.80 ± 6.21 mL, which was higher than that of the CaO_CS treatment group, while the difference in methane yield between the two groups was not significant. H_CS and HU_CS groups showed essentially identical methane production trends and yields. The DES_CS experimental group reached its maximum daily methane production of 476.63 ± 13.23 on the sixth day of anaerobic digestion. Although the methanogenesis process of the DES_CS group initiated slowly, and its cumulative methane production during the first five days was far lower than that of all other treatment groups; the cumulative methane production of this group was the highest at the end of anaerobic digestion, reaching 2852.55 ± 67.98 mL. DES pretreatment was found to exert a significant positive effect on the enhancement of methane yield from corn stover [18,28,29]. The methane yield was recorded as 356.67 ± 8.50 mL/g VS, with an improvement rate of 55.46%. Although methane production could also be increased by other pretreatment approaches, the methane yields of only CaO_CS (285.90 ± 5.81 mL/g VS) and BC_CS (301.48 ± 0.75 mL/g VS) were higher than that of synthetic mixture group MXL (263.81 ± 14.54). The methane yield from raw corn stover (229.08 ± 5.78 mL/g VS) was also lower than MXL. In conclusion, the complex structure of lignocellulose could be effectively degraded by deep eutectic solvents. More fermentable sugars were released for utilization by acidogenic bacteria. Accordingly, the substrate conversion rate was enhanced, and the maximum utilization of all components in biomass waste was achieved [30]. Furthermore, higher lignin removal and cellulose retention rates (Table 4) were proven to facilitate methanogenesis [28,31]. Notably, the DES pretreatment achieved a substantial enrichment of cellulose to 76.57%, which was significantly higher than that of the raw corn stover (30.89%), demonstrating its effectiveness in fractionating lignocellulosic components.

The cumulative methane yield varied significantly among groups (*p* < 0.001). DES_CS showed the highest yield (356.57 ± 8.50 mL/g VS), which was significantly higher than most groups (*p* < 0.05) but not different from XY and MCXY (*p* > 0.05). In contrast, the untreated CS group only achieved 229.03 ± 5.78 mL/g VS, significantly lower than all pretreated CS groups except BS_CS (Table 5).

### 3.2. Kinetics Analysis

According to results of experimental groups, the cumulative specific methane yields of H_CS, HU_CS, BS_CS, CaO_CS, BC_CS, and DES_CS were 271.73 ± 0.09, 270.41 ± 4.08, 251.52 ± 7.94, 285.90 ± 5.81, 301.48 ± 0.75, and 356.57 ± 8.50, respectively. Accordingly, compared with the raw CS group, the corresponding improvement rates reached 18.62%, 18.04%, 9.08%, 24.80%, 31.60%, and 55.46%, respectively. This study adopted the Modified Gompertz model and the Cone model to fit the cumulative methane production (Figure S1) [32]. As shown in Table 6, the coefficient of determination R^2^ of the Modified Gompertz model was mainly distributed in the range of 95.12% to 99.85%, while the R^2^ of the Cone model was higher than 98.94%. Based on Modified Gompertz and Cone model fitting results, distinct differences in maximum methane potential (*B*_0_) existed across groups. The overall maximum methane production potential of the system containing hemicellulose was higher, while the introduction of lignin would inhibit the realization of methane production potential. The results from both fitting models confirmed that DES pretreatment could increase the maximum methane production potential of corn stover by nearly 58%, demonstrating a remarkable improvement effect. The maximum methane production rate *R*max was regarded as a key indicator reflecting the rapid conversion capacity of substrates. The highest *R*max of 169.14 ± 10.26 mL/g VS/d was achieved by the XY group in the Modified Gompertz model, which was markedly higher than those of other groups. Hemicellulose-containing groups showed superior substrate conversion capacity. DES and CaO pretreatments effectively improved the methane production rate of corn stover. The lag phase *λ* was used to reflect the adaptation time of anaerobic microorganisms to substrates [33]. The lag phase λ of groups XY, XYLI, and MCXY was generally shorter, and was significantly lower than that of the experimental groups without hemicellulose, which confirmed that hemicellulose was readily utilized by anaerobic microorganisms. In contrast, the lag phase of the lignin-supplemented system was significantly prolonged. The lag phase of the DES_CS group reached 2.32 days, which was significantly longer than that of the other pretreatment groups, the MC group, and the MCLI group.

The hydrolysis rate constant k in the Cone model was used to characterize the substrate degradation rate [34]. Relatively high *k* values were recorded for XY, XYLI, and MCXY, which were 0.93 d^−1^, 0.78 d^−1^, and 0.41 d^−1^, respectively. The *k* value of MC was 0.26 d^−1^, which was lower than that of XY, indicating that the hydrolysis and conversion rate of cellulose was lower than that of hemicellulose. *k* values of both CaO_CS and BC_CS groups were 0.40 d^−1^ and 0.40 d^−1^, which were higher than the 0.21 d^−1^ of raw CS. The above results indicated that appropriate pretreatment methods would effectively enhance the hydrolysis rate of corn stover, thereby improving its methanogenic performance. Notably, the *k* value of DES_CS was 0.18 d^−1^, which was lower than that of CS, while its *B*_0_ value was found to be the highest among all tested groups. The time reached 95% of the maximum methane yield (T95) was 12 days for MCXY, and 14 days for MXL. Compared with the CS group, the T95 time of CaO_CS was significantly shortened, while the fermentation cycles of H_CS, HU_CS, and BS_CS groups were significantly prolonged.

### 3.3. Substance Conversion

**Figure 2:**
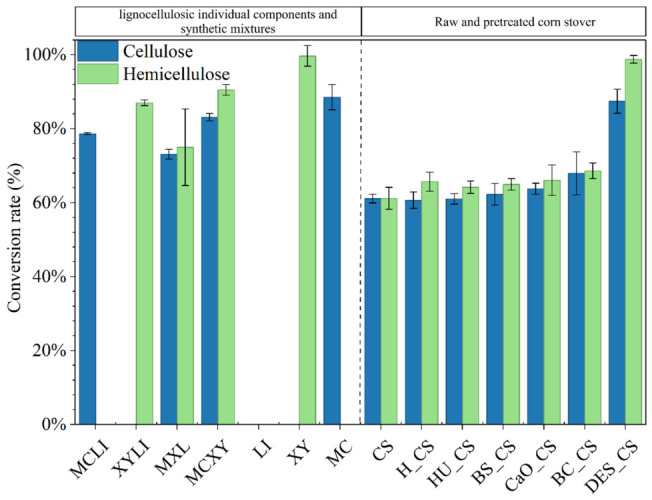
Conversion rates of cellulose and hemicellulose in different groups.

Hemicellulose conversion rate of 99.67% ± 2.78% was achieved in the XY group, while the cellulose conversion rate in the MC treatment was recorded as 88.53% ± 3.44%. Consistent with previous findings, cellulose and hemicellulose were confirmed to be easily biodegradable fractions that could be efficiently converted into biomethane by anaerobic microorganisms [10,27]. The conversion efficiencies of cellulose and hemicellulose across various groups are presented in Figure 2. The substrate conversion rates of lignocellulosic individual components and synthetic mixtures were generally found to be higher than that of corn stover with pretreatment, indicating that the complex intrinsic structure of raw corn stover significantly restricted the bioaccessibility of cellulose and hemicellulose [35]. Cellulose conversion rate of 78.65% ± 0.26% was obtained in MCLI, and a hemicellulose conversion rate of 86.99% ± 0.78% was determined in XYLI. These results demonstrated that individual components could be utilized sufficiently by anaerobic microbes in the absence of lignin embedding and structural barriers. Further comparison of mixed systems revealed that the cellulose and hemicellulose conversion rates in the MXL treatment were 73.07% ± 1.36% and 74.99% ± 10.36%, respectively, which were lower than those of the corresponding monomer systems. It might be implied that substrate competition or inter-component antagonism might be induced after blending different lignocellulosic fractions, thereby inhibiting the degradation of certain polysaccharide components.

By contrast, the conversion rates of cellulose and hemicellulose in the MCXY system were increased to 83.11% ± 1.00% and 90.53% ± 1.44%, respectively, both of which were higher than those in MXL. Lignin was identified as the critical factor affecting substrate conversion efficiency during anaerobic digestion. It was further validated that polysaccharide fractions exhibited greater degradability under purified conditions or with reduced structural resistance [28]. In raw corn stover, the conversion rates of cellulose and hemicellulose were only 61.10% ± 1.17% and 61.15% ± 2.98%, respectively, which were markedly lower than those of most monomer and mixed systems. The lignin barrier, cellulose crystalline regions, and the integrated cellulose–hemicellulose–lignin structure in raw corn stover were confirmed to greatly limit the accessibility of microorganisms and hydrolases to organic substrates. The conversion efficiencies of straw components were improved to varying degrees by different pretreatment methods, although distinct enhancement effects were observed. After H_CS, HU_CS, BS_CS, and CaO_CS pretreatments, cellulose conversion rates were 60.64% ± 2.21%, 61.01% ± 1.43%, 62.23% ± 2.91%, and 63.78% ± 1.51%, respectively, with only limited improvement shown relative to untreated CS. The corresponding hemicellulose conversion rates were 65.67% ± 2.57%, 64.15% ± 1.69%, 64.95% ± 1.54%, and 66.05% ± 4.13%, which were slightly higher than that of raw CS. It was indicated that these pretreatments promoted hemicellulose release and structural loosening to a certain extent, whereas the degradation of cellulose was not remarkably improved. In comparison, superior component conversion performance was exhibited by BC_CS and DES_CS. Specifically, the cellulose and hemicellulose conversion rates of BC_CS were 67.89% ± 5.81% and 68.61% ± 2.08%, approximately 6.80% and 7.46% higher than those of untreated CS. The most prominent improvement was detected in the DES_CS group, where cellulose and hemicellulose conversion rates reached 87.43% ± 3.25% and 98.74% ± 1.04%, with increases of 26.33% and 37.59% compared with raw CS. These findings demonstrated that the compact intrinsic structure of corn stover could be effectively destroyed by DES pretreatment, and the bioaccessibility of cellulose and hemicellulose was greatly enhanced, especially for hemicellulose degradation. Notably, the conversion level of DES_CS was close to or even exceeded that of several monomer mixed systems. It was suggested that component separation and delignification could be realized via DES pretreatment of lignocellulosic biomass, by which the structural barriers of raw straw were largely eliminated, allowing pretreated corn stover to display degradation potential comparable to pure component systems.

**Figure 3:**
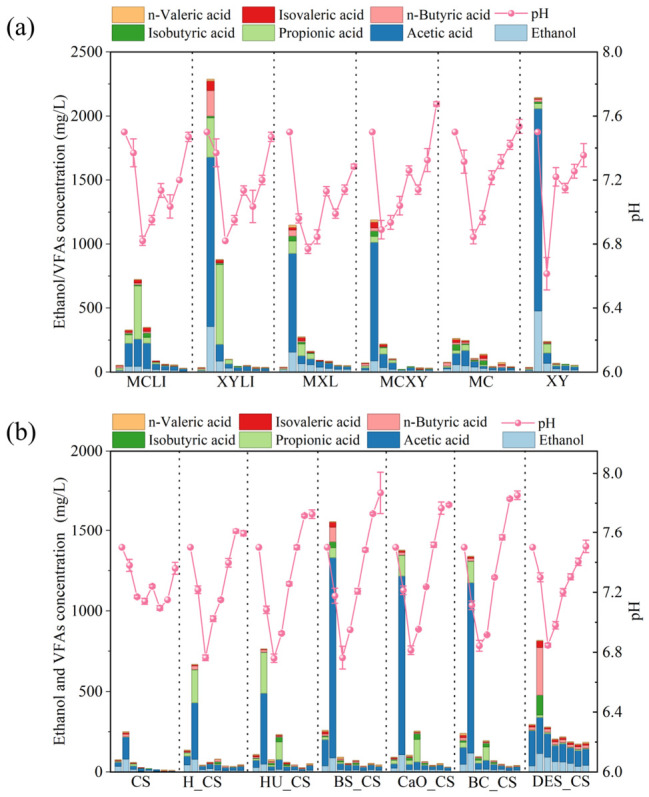
Ethanol/VFAs concentration and pH value in lignocellulosic individual components AD groups (**a**) and corn stover anaerobic digestion groups (**b**). The concentrations of ethanol, volatile fatty acids (VFAs), pH value and ammonia nitrogen were determined at anaerobic digestion days of 0, 1, 4, 8, 12, 16, 20, and 25. Error bars represent the standard deviations of triplicate measurements.

The physicochemical properties and assembly modes of substrate components significantly influenced the acidogenic potential. Alkaline lignin group was not included in the VFAs and pH analysis due to the absence of methane production during anaerobic digestion. Specifically, the peak concentrations of ethanol and total volatile fatty acids (TVFA) in the XY and XYLI groups were substantially higher than those in the MC and MCLI groups, accompanied by a more rapid decline in pH, confirming that hemicellulose is more susceptible to hydrolysis than cellulose (Figure 3). The significantly higher acetate concentration in the MCXY group compared to the MXL group in the early stage suggested that the presence of lignin exerted a shielding effect that inhibits the hydrolysis and acidogenesis efficiency of polysaccharides. Regarding metabolite profiles, lignin-containing groups exhibited a more pronounced accumulation of propionic acid during the initial stage. The marked differences in pH and VFAs between MXL and CS further corroborated the natural recalcitrance of lignocellulose. Notably, various pretreatments effectively bridged this gap, significantly enhancing the hydrolysis efficiency of CS by overcoming structural barriers. The early-stage VFA accumulation in these groups primarily originated from the extensive degradation of hemicellulose and partial cellulose. Compared with the acetate-dominated metabolite profiles obtained from CaO_CS and BC_CS groups, remarkable accumulations of butyric and valeric acids were found in the DES_CS group. It was confirmed that the compact lignocellulosic structure was efficiently destroyed by deep eutectic solvent pretreatment, and the formation of long-chain volatile fatty acids was also facilitated during the anaerobic process.

**Figure 4:**
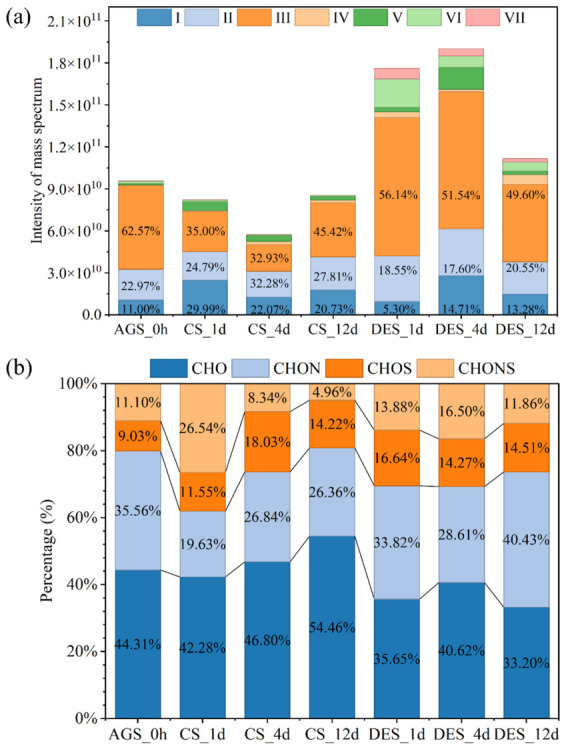
Absolute intensity of mass spectrum of DOM (**a**), and DOM element composition basing on relative intensity (**b**) at different anaerobic digestion periods for CS and DES groups. Relative proportions of lipids (I), aliphatic/proteins (II) and lignin/CRAM_like (III) were presented in the Figure 4a bar chart. AGS_0h represents the initial anaerobic granular sludge. To avoid confusion between abbreviations and improve the readability of the results, DES_time is used to represent the DES preprocessing experimental group at a specific time point.

The significant enhancement in the conversion of cellulose and hemicellulose was exhibited by DES pretreatment. It was indicated that the structural restriction of lignocellulose could be effectively eliminated by DES. The AD of lignocellulosic biomass was found to occur mainly within the first 12 days. Accordingly, to further reveal the transformation characteristics of organic substances before and after DES pretreatment, the molecular characteristics of dissolved organic matter (DOM) during DES pretreatment were determined on the first, fourth, and 12th day of anaerobic digestion (Figure S2). Seven categories prevalent in natural DOM were classified based on H/C and O/C ratios [36]: Lipids (I) for H/C = 1.5–2.0, O/C = 0–0.3; aliphatic/proteins (II) for H/C = 1.5–2.2, O/C = 0.3–0.67; lignin/carboxylic rich alicyclic molecules (CRAM-like) (III) for H/C = 0.7–1.5, O/C = 0.1–0.67; carbohydrates (IV) for H/C = 1.5–2.4, O/C = 0.67–1.2; unsaturated hydrocarbons (V) for H/C = 0.7–1.5, O/C = 0–0.1; aromatic structures (VI) for H/C = 0.2–0.7, O/C = 0–0.67; and tannin (VII) for H/C = 0.6–1.5, O/C = 0.67–1. The release and transformation pathways of soluble organic matter during the anaerobic digestion of corn stover were significantly altered by DES pretreatment. According to the analysis of DOM molecular composition, remarkable differences were observed in the molecular and chemical characteristics of DOM between the raw CS and DES systems at different digestion stages (Figure 4b). In the initial inoculum (AGS_0h), the proportion of CHO compounds was 44.31%, while CHON, CHOS, and CHONS were accounted for 35.56%, 9.03%, and 11.10%, respectively. It was demonstrated that DOM in the initial inoculum was mainly composed of oxygen and nitrogen containing organic compounds. The proportion of CHO compounds in the CS group was increased from 42.28% (CS_1d) to 54.46% (CS_12d), and the proportion of CHON compounds was elevated from 19.63% to 26.36%. It was implied that more oxygen and nitrogen containing organic molecules were gradually released during the degradation of untreated corn stover. In contrast, the overall proportion of CHO compounds was relatively lower in the DES group, recorded as 35.65% (DES_1d), 40.62% (DES_4d), and 33.20% (DES_12d). Meanwhile, the proportion of CHON compounds was increased to 40.43% at DES_12d, which was markedly higher than the 26.36% observed at CS_12d. It was suggested that the release and accumulation of nitrogen-containing organic components or microbial metabolites could be facilitated by DES pretreatment, and the transformation of DOM composition from simple oxygen-containing carbohydrates to complex nitrogen-containing organic molecules was driven.

The molecular diversity of DOM was significantly improved by DES treatment. The molecular formula numbers were recorded as 5147, 4275, and 6053 for CS_1d, CS_4d, and CS_12d, respectively. In addition, those of DES_1d, DES_4d, and DES_12d were reached 9941, 10,446, and 7936, which were generally much higher than those of the CS group (Table S1). It was demonstrated that the depolymerization of the complex structure of corn stover was effectively promoted by DES pretreatment, and more detectable DOM molecules were released. Lignin/CRAM-like compounds (III) were identified as the most dominant DOM components with 62.57% in AGS_0h (Figure 4a). In the CS group, it decreased to 35.00% at CS_1d, maintained at 32.93% at CS_4d, and subsequently rebounded to 45.42% at CS_12d. It was inferred that partial lipids, proteins, and easily degradable organic matter were released or transformed by untreated straw in the early anaerobic stage. As digestion proceeded, recalcitrant lignin-like and CRAM-like substances were gradually formed as the major residual DOM components. In the DES group, lignin/CRAM-like compounds were maintained at consistently high levels of 56.14% (DES_1d), 51.54% (DES_4d), and 49.60% (DES_12d), which were significantly higher than those of the CS group at corresponding time points. It was indicated that the lignocellulosic structure of corn stover could be effectively destroyed by DES pretreatment, and lignin-like aromatic compounds were promoted to enter the liquid-phase DOM. The molecular distribution of DOM revealed the potential mechanism by which component release and anaerobic digestion performance of corn stover were improved by DES pretreatment at the molecular level. The lignocellulose deconstruction and DOM molecular diversity were enhanced by DES, whereby a richer substrate basis was provided for subsequent hydrolysis, acidification, and methanogenesis processes [37,38].

### 3.4. Microbial Community Composition

**Figure 5:**
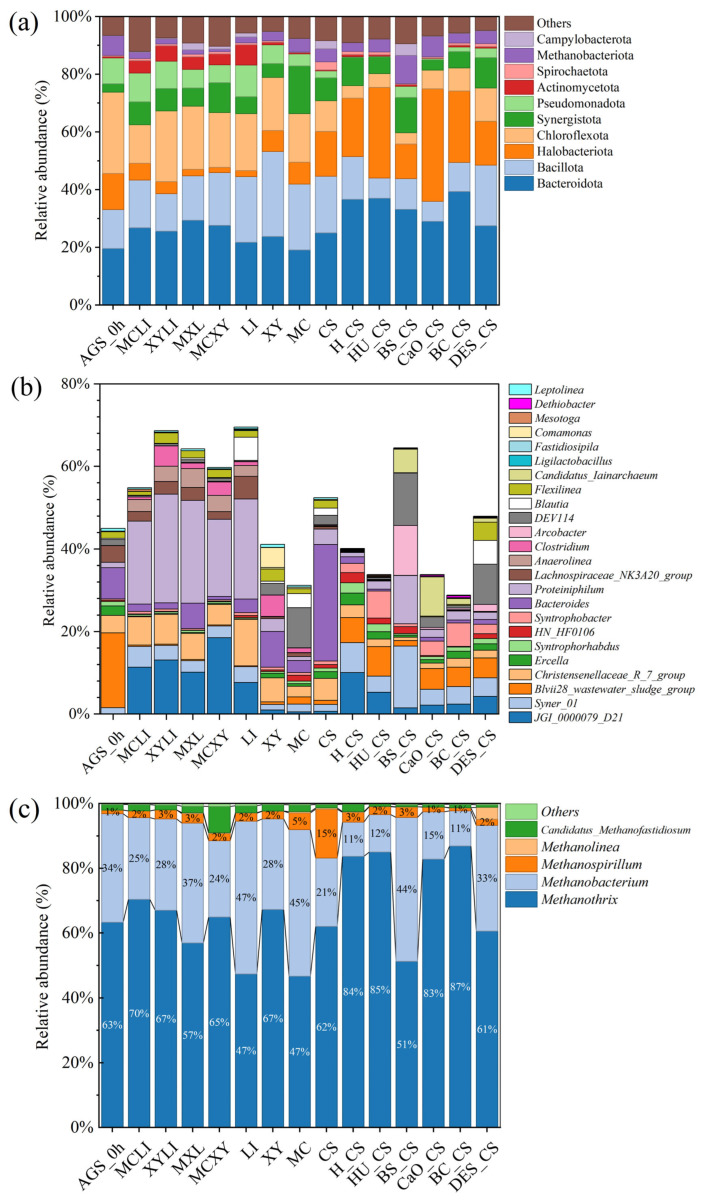
Microbial community composition in different anaerobic digestion groups at the phylum and genus levels. Relative abundance of the top 10 microbial phyla (**a**), top 25 bacterial genera (**b**), and top 5 archaeal genera (**c**).

At the end of AD in each experimental group (25th day), samples were collected to determine the structure of microbial communities (Figure 5). As shown in Figure 5a, the microbial community composition at the phylum level varied among the different anaerobic digestion groups. The dominant phyla were mainly Bacteroidota, Bacillota, Halobacterota, Chloroflexota, Synergistota, and Pseudomonadota. Notably, Bacteroidota and Bacillota were the most abundant phyla in most samples (Figure 5a). Among all the pretreated CS groups, the DES_CS group exhibited the highest richness and diversity indices (Chao1 = 759, Shannon = 6.87), indicating that DES pretreatment was more conducive to the formation of more diverse microbial communities compared with other treatments (Table S2). Lignin-containing systems (MCLI, XYLI, MXL, LI) were dominated by complex substrate-degrading bacteria such as *Proteiniphilum* and *Christensenellaceae_R_7_group*, whereas fast-growing fermentative bacteria (*Bacteroides*) were enriched in cellulose/hemicellulose-only groups (MC, XY). As a typical genus involved in carbohydrate degradation [27], *Bacteroides* was markedly enriched (28.26%) in the CS group, while it only accounted for 1.15% in the DES_CS group (Figure 5b). Meanwhile, its relative abundance in the XY group reached 8.66%. The proliferation of Bacteroides in the CS group was driven by the retention of large amounts of readily degradable carbohydrates, whereas other pretreatments likely removed part of the soluble sugars to varying degrees. In addition, the high accessibility and rapid degradation of xylan also accounted for its enrichment in the XY group. Significant differences were observed between the microbial community of the synthetic ternary system (MCXYLI) and raw corn stover (CS). The structure of the corn stover-associated microbial community was significantly reshaped by DES pretreatment, which led to the enrichment of *Syntrophobacter*, *DEV114*, *JGI_0000079_D21*, *Syner_01*, and *Blvii28_wastewater_sludge_group*. Such shifts in microbial community structure appeared to be beneficial for enhancing methane production. Specifically, compared with the MC and CS groups, syntrophic bacteria (*Syner_01*) and methanogenic archaea tended to be significantly enriched in the DES_CS group. This enrichment likely fostered a more robust microbial metabolic interaction network, which could provide a reasonable biological foundation for the elevated methane yield. Spearman correlation analysis showed that key hydrolytic bacteria (*Clostridium, Leptolinea*) and syntrophic bacteria (*Syntrophobacter*) were significantly positively correlated with cellulose and hemicellulose conversion rates, indicating their critical roles in promoting lignocellulose degradation (Figure S4).

As shown in the archaeal community structure diagram (Figure 5c), *Methanothrix* and *Methanobacterium* were the dominant methanogenic genera across all digesters. *Methanothrix*, the acetotrophic methanogen, produced methane via acetate cleavage, and was the primary acetate-utilizing methanogen in anaerobic digestion systems [39]. The relative abundance of *Methanothrix* was extremely high (83%~87%) in the H_CS, HU_CS, CaO_CS and BC_CS groups, while it reached 51.23% in the BS_CS group, 60.58% in the DES_CS group, and 62.00% in the CS group. In the lignocellulosic individual components and synthetic mixtures experimental groups, the relative abundances of *Methanothrix* were 46.67% (MC), 67.27% (XY), and 56.94% (MXL), respectively, while the relative abundance of *Methanobacterium* increased to 45.23% (MC), 27.96% (XY), and 37.00% (MXL), compared to H_CS and others pretreatment groups. This result was consistent with the findings reported in previous studies [10,27,40]. Unrestricted by the complex lignocellulose structure, hydrogenotrophic and acetoclastic pathways could concurrently drive methanogenesis in single or mixed fermentations of cellulose, hemicellulose, and lignin.

### 3.5. Metagenomic Analysis of Metabolic Characteristics

Samples in CS and DES_CS groups, were collected on the first, fourth, 12th and 25th day of anaerobic fermentation for metagenomic sequencing. The microbial functions in both the CS and DES systems were indicated to be mainly concentrated in the Metabolism category by KEGG functional annotation results, with its absolute abundance being significantly higher than those of Genetic Information Processing, Environmental Information Processing, and Cellular Processes (Figure S3a,b). In CS group, the abundance of Metabolism was changed from 3.69 × 10^7^ in CS_1d to 3.66 × 10^7^ in CS_25d, with relatively small fluctuations being exhibited. In the DES group, the abundances of Metabolism in DES_1d, DES_4d, DES_12d, and DES_25d were recorded as 3.71 × 10^7^, 3.70 × 10^7^, 3.51 × 10^7^, and 3.69 × 10^7^, respectively, which were maintained at a relatively high level. It was indicated by this result that the total abundance of primary metabolic functions was not simply increased by DES pretreatment. The anaerobic conversion process was more likely to be influenced by regulating the composition of specific metabolic pathways. At the Level 2 functional level, Carbohydrate metabolism, Amino acid metabolism, and Energy metabolism were identified as the most prominent functional categories (Figure S3c,d). In the CS group, the abundance of Carbohydrate metabolism was maintained between 4.55 × 10^6^ and 4.76 × 10^6^ from CS_1d to CS_25d. It was implied that the polysaccharide components in raw corn stover were slowly released and continuously participated in microbial metabolism. In the DES group, the abundances of Carbohydrate metabolism in DES_1d, DES_4d, DES_12d, and DES_25d were 4.62 × 10^6^, 4.54 × 10^6^, 4.37 × 10^6^, and 4.64 × 10^6^, respectively, showing a trend of initial decline followed by recovery. This trend was related to the rapid consumption of easily degradable carbon sources in the early stage and the continuous release of residual polysaccharides in the later stage after DES pretreatment. Notably, the abundance of Amino acid metabolism in DES_25d was reached 3.12 × 10^6^, which was higher than the 2.78 × 10^6^ in CS_25d. It was suggested that a stronger function in the conversion of nitrogen-containing organic matter and protein-like substances was possessed by the DES system in the later stage. Combined with the DOM analysis results showing that the proportion of CHON molecules was increased in the later stage of the DES group, it was inferred that the deconstruction of lignocellulose was promoted by DES pretreatment, and the release of nitrogen-containing organic matter as well as the composition of microbial metabolic products were also altered.

**Figure 6:**
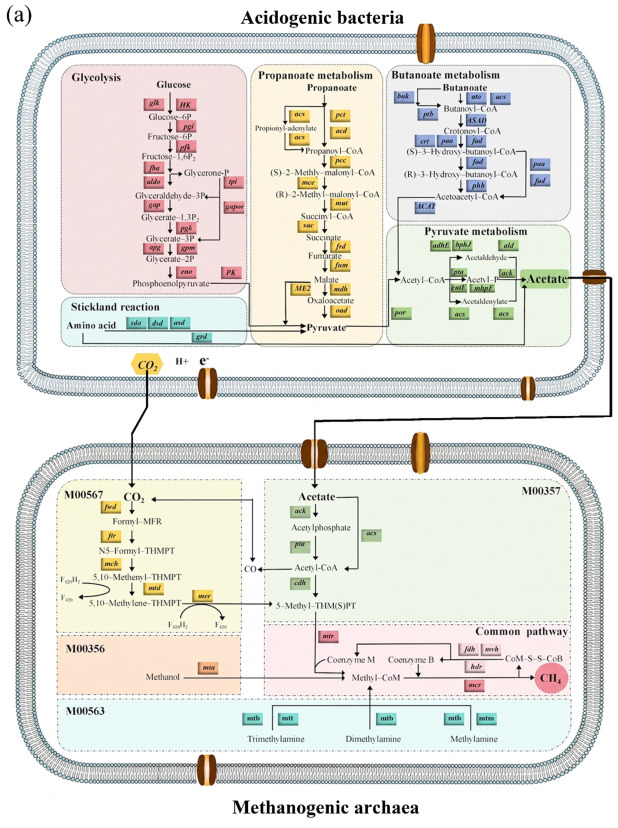

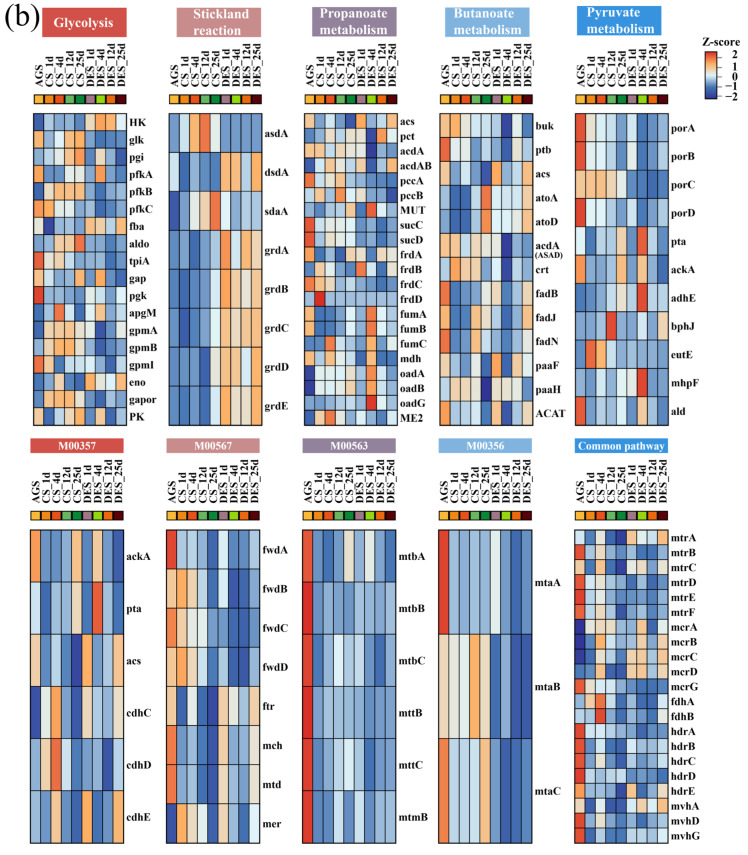
Multiple group heatmap of relative abundances of functional genes (**a**), and key metabolic pathways of acidogenesis phase and methanogenesis phase (**b**) at different anaerobic digestion periods for CS and DES groups. AGS represents the initial anaerobic granular sludge.

As shown in Figure 1b, the DES pretreatment group exhibited a characteristically lagged yet robust methane production pattern. The initial low yield was likely not only due to microbial acclimatization but also reflected a metabolic preparation phase required to utilize DES-modified lignocellulosic substrates. After the third day, a significant metabolic shift occurred. The KEGG functional annotation (Figure 6) revealed a concerted up-regulation of key glycolysis genes (*HK* and *PK*). This enrichment suggested a rapid flux of carbon towards pyruvate, providing a massive influx of intermediates for subsequent methanogenesis. Furthermore, the concurrent enrichment of *pta*, *adhE*, *ackA*, and *mhpF* indicated a highly active pyruvate-to-acetate/ethanol pathway. These genes were integral to substrate degradation and methanogenic pathways [31]. The co-enrichment of these genes suggested that DES pretreatment facilitated a more efficient syntrophic interaction between acidogens and methanogens. Specifically, the pta-ackA pathway optimized the conversion efficiency of Acetyl-CoA to acetate, directly enriching the substrate pool for acetoclastic methanogenesis. Therefore, the observed surge in methane production after the fourth day was the macro-level manifestation of this microbial metabolic reprogramming, in which the synergy between substrate accessibility and functional gene expression levels was fully achieved.

## 4. Conclusions

This study provided a detailed comparative analysis of the methane yield, substance conversion, and microbial communities among lignocellulosic individual components, synthetic mixtures, and differently pretreated corn stover during anaerobic digestion. The results demonstrated that the methane yields of cellulose and hemicellulose reached 320.81 ± 1.85 mL/g VS and 352.70 ± 6.58 mL/g VS, respectively. The synthetic mixture of cellulose and hemicellulose (MCXY) exerted a positive promoting effect on biomethane production, with a synergistic effect index of 101.51%. Lignin did not produce biogas in AD system, and its presence had an inhibitory effect on the methanogenesis of cellulose and hemicellulose, especially that of hemicellulose. The cumulative methane production of XYLI decreased by 8.55%, accompanied by a marked reduction in the synergistic effect index. Notably, pretreatment significantly improved the methane production potential of corn stover. DES_CS achieved the highest methane yield of 356.57 ± 8.50 mL/g VS, which was 55.46% higher than that of the untreated group. DES pretreatment deconstructed lignocellulosic matrix and distinctly increased DOM molecular diversity, thus providing superior substrate conditions for improving anaerobic digestion performance. Microbial community analysis revealed that DES pretreatment significantly reshaped the bacterial structure, enriching syntrophic taxa over the carbohydrate-degrading *Bacteroides* found in raw corn stover, thereby fostering a more robust metabolic network for methane production. The metabolic activities of hydrogenotrophic and acetoclastic methanogenic archaea proceeded simultaneously.

## Figures and Tables

**Table 1:** Characteristics of substrates and inoculum.

Characteristics	Anaerobic Granular Sludge	Alkaline Lignin	Microcrystalline Cellulose	Xylan	Corn Stover
TS (%) ^a^	8.74 ± 0.01	91.92 ± 3.01	96.23 ± 0.01	95.83 ± 0.87	92.33 ± 1.37
VS (%) ^a^	5.96 ± 0.08	84.71 ± 0.68	96.20 ± 0.01	95.79 ± 0.86	84.71 ± 0.96
NH_3_-N (mg/L)	784 ± 2.36	N.A.	N.A.	N.A.	N.A.
pH	7.96 ± 0.01	N.A.	N.A.	N.A.	N.A.
Lignin ^b^ (%)	0.68 ± 0.03	100.00	0.00	0.00	4.23 ± 0.07
Cellulose ^b^ (%)	1.64 ± 0.09	0.00	100.00	0.00	30.89 ± 0.44
Hemicellulose ^b^ (%)	2.58 ± 0.13	0.00	0.00	100.00	24.23 ± 0.18
C (%) ^b^	38.86 ± 0.24	39.18 ± 0.04	43.39 ± 0.06	42.08 ± 0.05	39.18 ± 0.12
H (%) ^b^	5.48 ± 0.27	5.18 ± 0.30	6.41 ± 0.02	6.60 ± 0.03	5.18 ± 0.07
N (%) ^b^	8.74 ± 0.07	0.88 ± 0.04	0.00	0.01 ± 0.00	0.88 ± 0.04
O (%) ^b^	42.76 ± 0.38	41.99 ± 0.30	49.09 ± 0.01	50.98 ± 0.02	41.99 ± 0.21

^a^ based on wet weight, ^b^ based on dry weight, N.A. means Not Applicable.

**Table 2:** Experimental design of mixing ratio of synthetic mixtures.

Category	Mixing Method	Mixing Ratio ^a^
MCXY	Microcrystalline cellulose + Xylan	7:6
MCLI	Microcrystalline cellulose+ Alkaline lignin	7:1
XYLI	Xylan + Alkaline lignin	6:1
MXL	Microcrystalline cellulose + Xylan + Alkaline lignin	7:6:1
LI	Only Alkaline lignin	N.A.
MC	Only Microcrystalline cellulose	N.A.
XY	Only Xylan	N.A.

^a^ based on total solid, N.A. means Not Applicable.

**Table 3:** Pretreatment methods and parameters of corn stover.

Category	Methods	Dosage of Reagent	Solid-to-Liquid Ratio ^a^	Temperature (°C)	Treat Time (h)
CS	N.A.	N.A.	N.A.	N.A.	N.A.
H_CS	Thermal hydrolysis	N.A.	1:6	90	2
HU_CS	Hydrothermal-urea co-pretreatment	6% CO(NH_2_) (TS) ^b^	1:6	90	2
BS_CS	Biogas slurry pretreatmet	Biogas slurry	1:4 ^c^	35	72
CaO_CS	CaO pretreatment	10%CaO (TS) ^b^	1:4 ^d^	35	72
BC_CS	Biogas slurry-CaO co-pretreatment	10%CaO (TS) ^b^	1:4 ^c^	35	72
DES_CS	Deep eutectic solvent pretreatment	Choline chloride: lactic acid = 1:3 (molar ratio)	1:15	130	3

^a^ Solid-to-liquid ratio based on mass, ^b^ based on corn stover total solid, ^c^ Adjust the total solid (TS) to 20% using biogas slurry, ^d^ Adjust TS to 20% using distilled water, N.A. means Not Applicable.

**Table 4:** Compositions of lignocellulose in corn stover after pretreatment in different groups.

Category	Lignin (% TS) ^a^	Cellulose (% TS) ^a^	Hemicellulose (% TS) ^a^
CS	4.23 ± 0.07	30.89 ± 0.44	24.23 ± 0.18
H_CS	3.22 ± 0.67	32.71 ± 0.11	28.97 ± 0.06
HU_CS	3.17 ± 0.08	33.85 ± 0.17	27.61 ± 0.12
BS_CS	3.15 ± 0.11	33.19 ± 0.09	26.65 ± 0.10
CaO_CS	1.20 ± 0.08	29.30 ± 0.08	13.79 ± 0.08
BC_CS	1.52 ± 0.09	28.09 ± 0.10	14.59 ± 0.07
DES_CS	4.47 ± 0.07	76.57 ± 0.07	12.25 ± 0.20

^a^ based on total solid.

**Table 5:** Cumulative specific methane yield (CSMY) and synergistic effect index (SEI) in experimental groups. Values are presented as mean ± standard deviation (n = 3). Different lowercase letters within the same column indicate significant differences among groups according to one-way ANOVA followed by Tukey’s post-hoc test (*p* < 0.05).

Groups	CSMY (mL/g VS)	CSMY Excluding Lignin (mL/g VS)	SEI (%)
MCXY	340.60 ± 10.94 ^a^	340.60 ± 10.94	101.51%
MCLI	278.49 ± 14.47 ^c^	318.27 ± 16.54	99.21%
XYLI	269.22 ± 6.69 ^c^	324.92 ± 7.81	89.05%
MXL	263.81 ± 14.55 ^c^	284.10 ± 15.67	84.67%
LI	0.00 ^e^	0.00	N.A.
XY	352.70 ± 6.58 ^a^	352.70 ± 6.58	N.A.
MC	320.81 ± 11.85 ^b^	320.81 ± 11.85	N.A.
CS	229.03 ± 5.78 ^d^	N.A.	N.A.
H_CS	271.73 ± 0.09 ^c^	N.A.	N.A.
HU_CS	270.41 ± 4.08 ^c^	N.A.	N.A.
BS_CS	251.52 ± 7.94 ^d^	N.A.	N.A.
CaO_CS	285.90 ± 5.82 ^c^	N.A.	N.A.
BC_CS	301.47 ± 0.77 ^b^	N.A.	N.A.
DES_CS	356.57 ± 8.50 ^a^	N.A.	N.A.

N.A., Not applicable.

**Table 6:** Kinetic analysis of methane yield.

Models	Modified Gompertz Model				Cone Model			
Parameters	*B*_0_ (mL/g VS)	*R*_max_ (mL/g VS/d)	*λ* (d)	R^2^ (%)	*B*_0_ (mL/g VS)	*k* (d^−1^)	R^2^ (%)	T95 (d)
MCXY	333.91 ± 21.87	62.67 ± 4.19	1.34 × 10^−3^ ± 1.04 × 10^−4^	98.87%	350.61 ± 18.49	0.41 ± 0.03	99.63%	12
MCLI	273.31 ± 15.52	43.77 ± 2.51	1.34 ± 0.07	96.44%	272.19 ± 12.38	0.31 ± 0.02	99.42%	13
XYLI	260.82 ± 13.09	117.03 ± 6.68	0.21 ± 0.01	97.62%	265.59 ± 17.57	0.78 ± 0.06	98.94%	10
MXL	255.07 ± 13.59	48.95 ± 2.71	0.55 ± 0.03	99.28%	262.81 ± 15.31	0.33 ± 0.03	99.71%	14
XY	338.10 ± 23.22	169.14 ± 10.26	0.10 ± 0.01	98.10%	346.85 ± 13.03	0.93 ± 0.07	99.41%	10
MC	313.41 ± 19.77	57.68 ± 2.94	1.13 ± 0.09	99.45%	321.18 ± 17.45	0.26 ± 0.02	99.83%	12
CS	224.99 ± 11.45	30.94 ± 1.80	4.98 × 10^−3^ ± 3.92 × 10^−4^	99.13%	244.37 ± 14.26	0.21 ± 0.02	99.36%	12
H_CS	265.74 ± 17.85	20.70 ± 1.39	1.07 × 10^−3^ ± 8.34 × 10^−5^	98.1%	287.44 ± 17.11	0.22 ± 0.02	99.85%	20
HU_CS	252.29 ± 16.07	30.74 ± 1.86	3.84 × 10^−3^ ± 3.05 × 10^−4^	95.35%	273.13 ± 15.54	0.21 ± 0.01	99.94%	20
BS_CS	249.29 ± 13.88	20.39 ± 1.35	2.73 × 10^−3^ ± 2.41 × 10^−5^	98.20%	232.28 ± 14.18	0.20 ± 0.01	99.15%	18
CaO_CS	281.79 ± 19.24	45.52 ± 2.70	2.16 × 10^−3^ ± 1.90 × 10^−4^	98.95%	301.20 ± 13.52	0.40 ± 0.03	99.65%	10
BC_CS	300.70 ± 17.58	32.08 ± 1.83	1.95 × 10^−4^ ± 1.32 × 10^−4^	95.12%	314.22 ± 20.51	0.40 ± 0.02	99.80%	13
DES_CS	354.98 ± 18.49	54.80 ± 2.77	2.32 ± 0.18	99.85%	354.56 ± 24.31	0.18 ± 0.02	99.65%	13

## Data Availability

The original contributions presented in this study are included in the article/Supplementary Materials. Further inquiries can be directed to the corresponding author.

## Supplementary Materials

The following supporting information can be downloaded at: https://www.mdpi.com/article/10.3390/bioengineering13060630/s1. Figure S1: Curve fitting of Modified Gompertz model (a,b), and Cone model (c,d) in lignocellulosic individual components and corn stover anaerobic digestion groups; Figure S2: VK diagram of DOM components at different time of anaerobic digestion system of initial anaerobic granular sludge (a), CS (b–d) and DES group (e–g). AGS_0h represents the initial anaerobic granular sludge. CS_1d, CS_4d and CS_12d correspond to days 1, 4 and 12 of anaerobic digestion of untreated corn stover, respectively. DES_1d, DES_4d and DES_12d indicate samples collected on days 1, 4 and 12 during anaerobic digestion of DES-pretreated corn stover; Figure S3: Absolute abundance of microbial functional pathways annotated at KEGG level 1 (a,b), level 2 (c,d) in CS and DES-pretreated corn stover AD groups; Figure S4: Correlation between microbial community composition and key fermentation performance indicators during anaerobic digestion. Spearman correlation analysis showing the associations between microbial genera and key anaerobic digestion performance indicators (methane yield, cellulose conversion rate, and hemicellulose conversion rate). The color of the lines indicates the direction of the correlation (purple: positive correlation; orange: negative correlation), and the line thickness represents the absolute value of the Spearman’s correlation coefficient (|r|). Solid lines denote significant correlations (*p* < 0.05), while dashed lines indicate non-significant correlations (*p* ≥ 0.05). The small squares on the left further illustrate the correlation coefficients (Spearman’s r) between each microbial genus and individual performance indicators, with red representing positive correlations and blue representing negative correlations. Structure, defined as 0 for lignocellulosic individual components and 1 for corn stover; Table S1: Molecular characterization of DOM in CK and DES-pretreated groups; Table S2: Alpha diversity indices of microbial communities in different substrate groups during anaerobic digestion.

## Abbreviations

The following abbreviations are used in this manuscript:

AD	anaerobic digestion
AGS	anaerobic granular sludge
BC_CS	biogas slurry-calcium oxide co-pretreated corn stover
BS_CS	biogas slurry pretreated corn stover
CaO_CS	calcium oxide pretreated corn stover
CHO	compounds containing only carbon, hydrogen and oxygen
CHON	compounds containing carbon, hydrogen, oxygen and nitrogen
CHONS	compounds containing carbon, hydrogen, oxygen, nitrogen and sulfur
CHOS	compounds containing carbon, hydrogen, oxygen, and sulfur
CS	corn stover
CSMP	cumulative specific methane yield
CRAM_like	carboxyl-rich alicyclic molecule-like
DES	deep eutectic solvent
DOM	dissolved organic matter
H_CS	thermally hydrolysis pretreated corn stover
HU_CS	thermally hydrolysis-urea co-pretreated corn stover
LI	alkaline lignin
MC	microcrystalline cellulose
MCLI	binary mixture of cellulose and lignin
MCXY	binary mixture of cellulose and hemicellulose (xylan)
MXL	ternary mixture of cellulose, hemicellulose (xylan) and lignin
TS	total solids
SEI	synergistic effect index
VS	volatile solids
VK diagram	Van Krevelen Diagram
XY	xylan
XYLI	binary mixture of hemicellulose (xylan) and lignin
