# Supplementary material for: Enhancing Biomethane Production from Corn Stover: Insights into Lignocellulosic Component Interactions and Pretreatment Efficacy
Source: Bioengineering (Basel). 2026 May 28;13(6):630. doi: 10.3390/bioengineering13060630 (PMC13295671; doi:10.3390/bioengineering13060630)
Supplement: Supplementary file 1 [file bioengineering-13-00630-s001.zip › bioengineering-4326904-supplementary.pdf]

Supplementary Materials

Enhancing Biomethane Production from Corn Stover: Insights into Lignocellulosic Component Interactions and Pretreatment Efficacy

Xiteng Chen, Lu Liu, Hairong Yuan and Xiujin Li *

Department of Environmental Science and Engineering, Beijing University of Chemical Technology, Beijing 100029, China; 2020400061@buct.edu.cn (X.C.)
* Correspondence: xjlibuct@163.com

There are three figures and two tables in this supplementary materials:

Fig S1. Curve fitting of Modified Gompertz model (a, b), and Cone model (c, d) in lignocellulosic individual components and corn stover anaerobic digestion groups

Fig S2. VK diagram of DOM components at different time of anaerobic digestion system of initial anaerobic granular sludge (a), CS (b, c, d) and DES group (e, f, g). AGS_0h represents the initial anaerobic granular sludge. CS_1d, CS_4d and CS_12d correspond to days 1, 4 and 12 of anaerobic digestion of untreated corn stover, respectively. DES_1d, DES_4d and DES_12d indicate samples collected on days 1, 4 and 12 during anaerobic digestion of DES-pretreated corn stover

Fig S3. Absolute abundance of microbial functional pathways annotated at KEGG level 1 (a)(b), level 2 (c)(d) in CS and DES-pretreated corn stover AD groups

Fig S4. Correlation between microbial community composition and key fermentation performance indicators during anaerobic digestion. Spearman correlation analysis showing the associations between microbial genera and key anaerobic digestion performance indicators (methane yield, cellulose conversion rate, and hemicellulose conversion rate). The color of the lines indicates the direction of the correlation (purple: positive correlation; orange: negative correlation), and the line thickness represents the absolute value of the Spearman's correlation coefficient (|r|). Solid lines denote significant correlations ($P < 0.05$), while dashed lines indicate non-significant correlations ($P \geqslant 0.05$). The small squares on the left further illustrate the correlation

coefficients (Spearman's r) between each microbial genus and individual performance indicators, with red representing positive correlations and blue representing negative correlations. Structure, defined as 0 for lignocellulosic individual components and 1 for corn stover.

Table S1. Molecular characterization of DOM in CK and DES-pretreated groups

Table S2 Alpha diversity indices of microbial communities in different substrate groups during anaerobic digestion

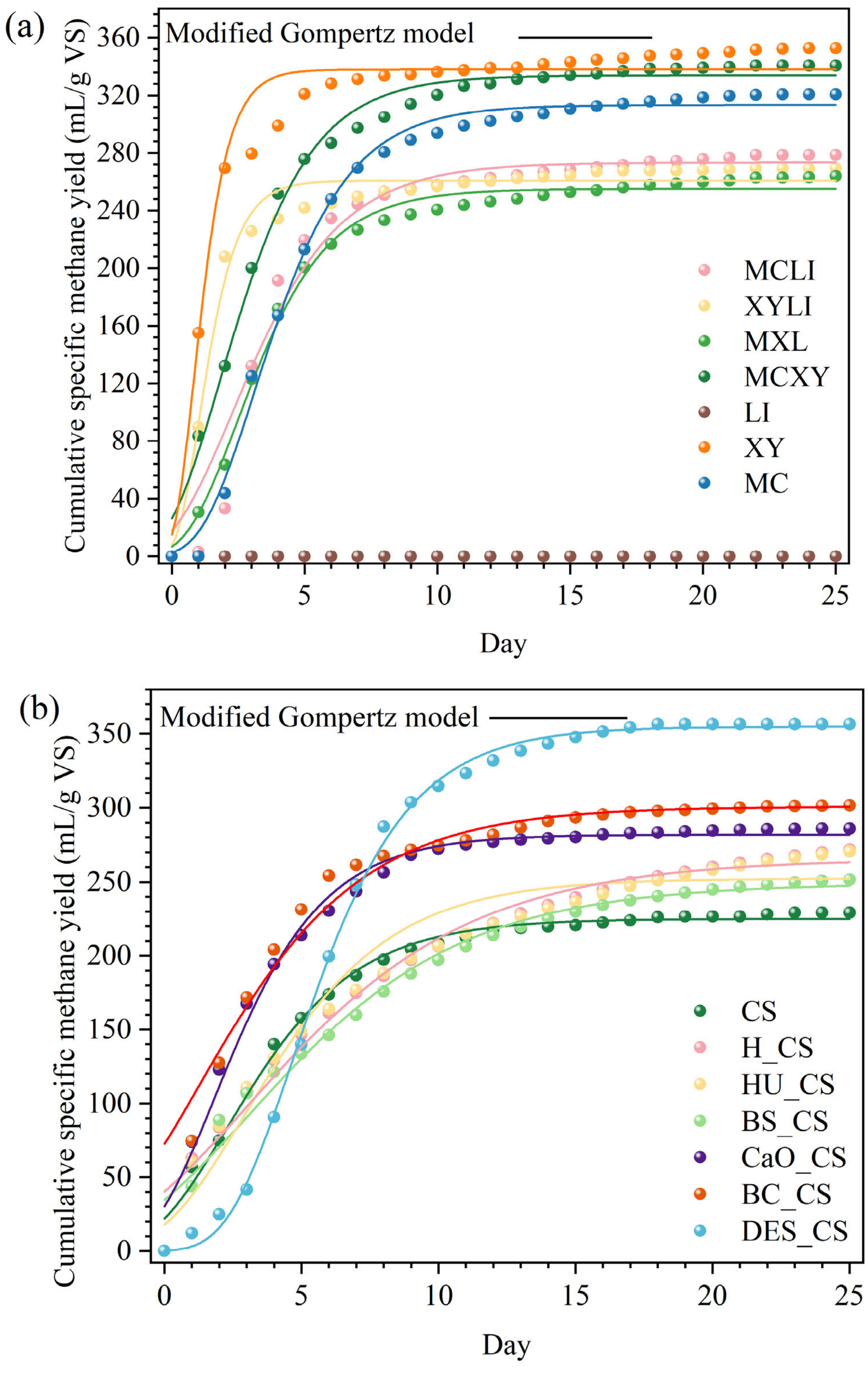

(a)
Modified Gompertz model
Cumulative specific methane yield (mL/g VS)
Day
MCLI
XYLI
MXL
MCXY
LI
XY
MC
(b)
Modified Gompertz model
Cumulative specific methane yield (mL/g VS)
Day
CS
H_CS
HU_CS
BS_CS
CaO_CS
BC_CS
DES_CS

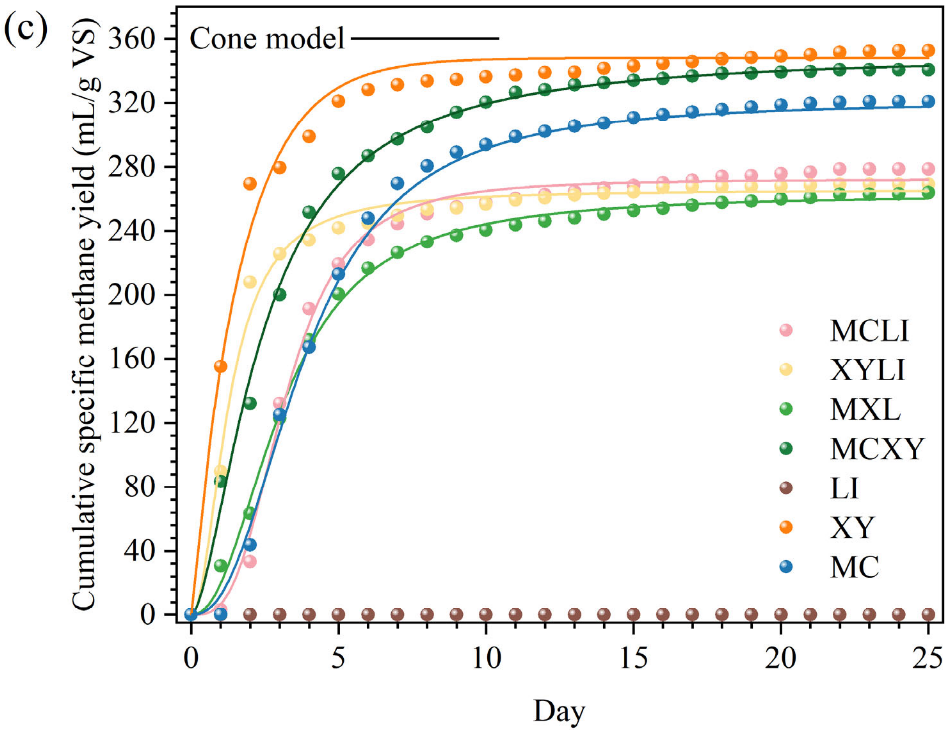

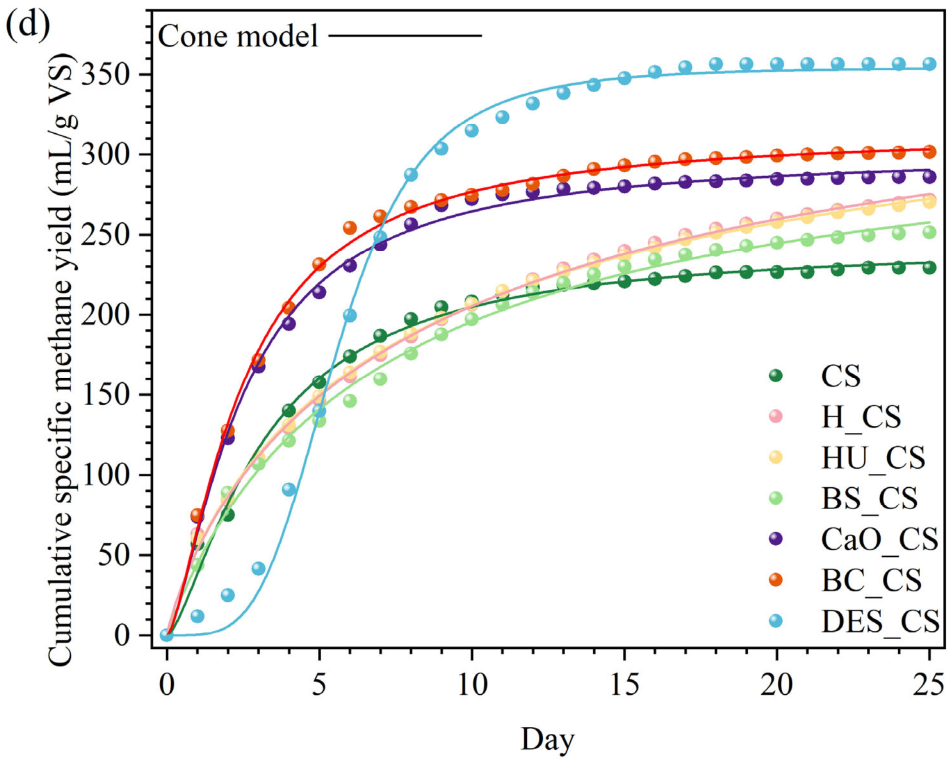

Fig S1. Curve fitting of Modified Gompertz model (a, b), and Cone model (c, d) in lignocellulosic individual components and corn stover anaerobic digestion groups

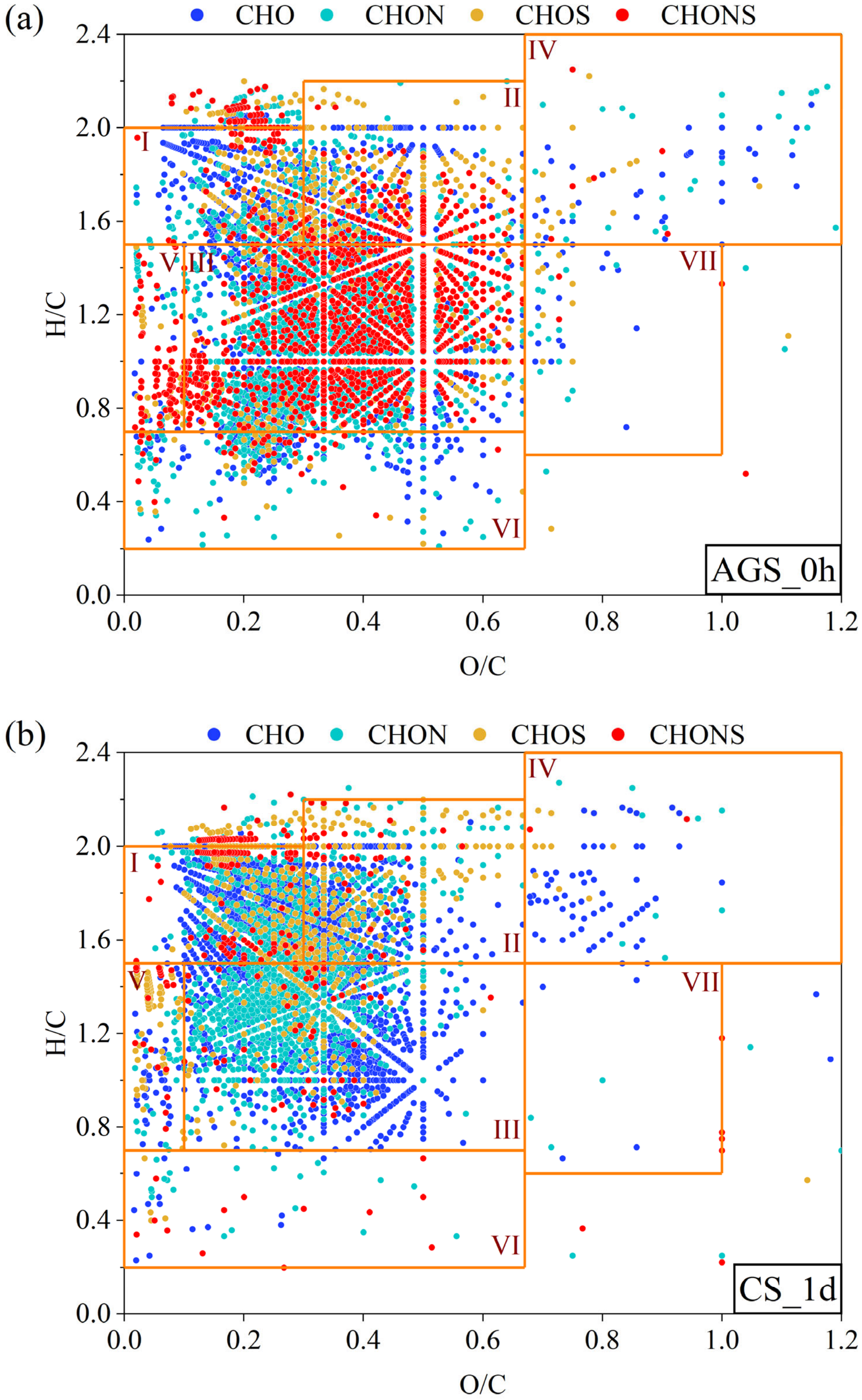

(a)
CHO
CHON
CHOS
CHONS
H/C
O/C
I
II
III
IV
V
VI
VII
AGS_0h
(b)
CHO
CHON
CHOS
CHONS
H/C
O/C
I
II
III
IV
V
VI
VII
CS_1d

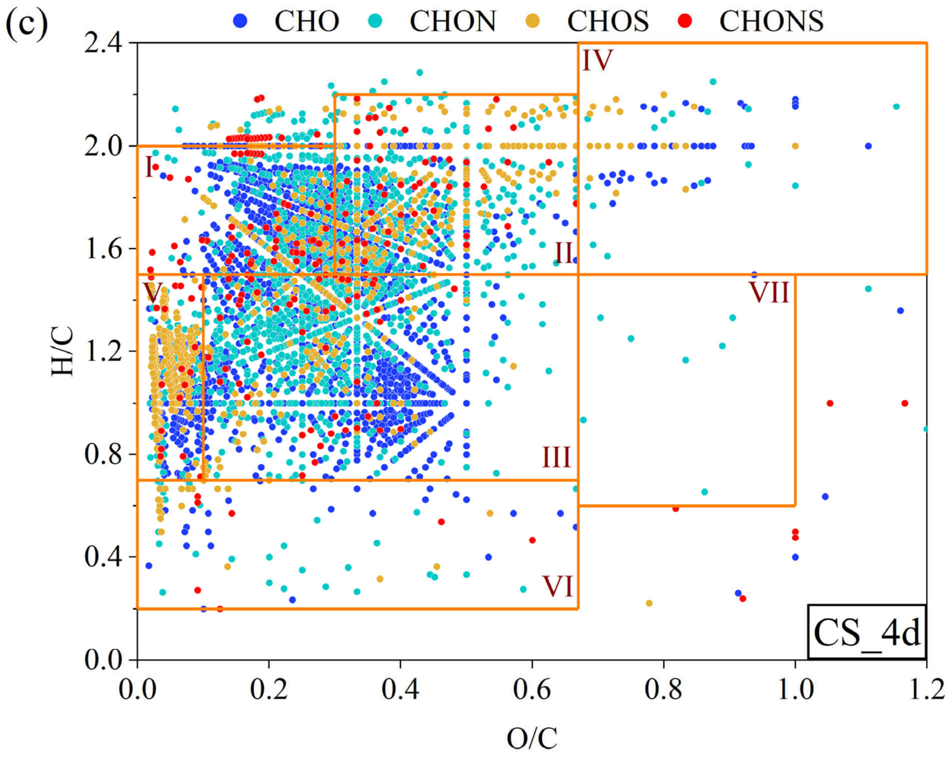
(c)
CHO
CHON
CHOS
CHONS
I
II
III
IV
V
VI
VII
CS_4d
H/C
O/C
0.0
0.4
0.8
1.2
1.6
2.0
2.4
0.2
0.6
1.0

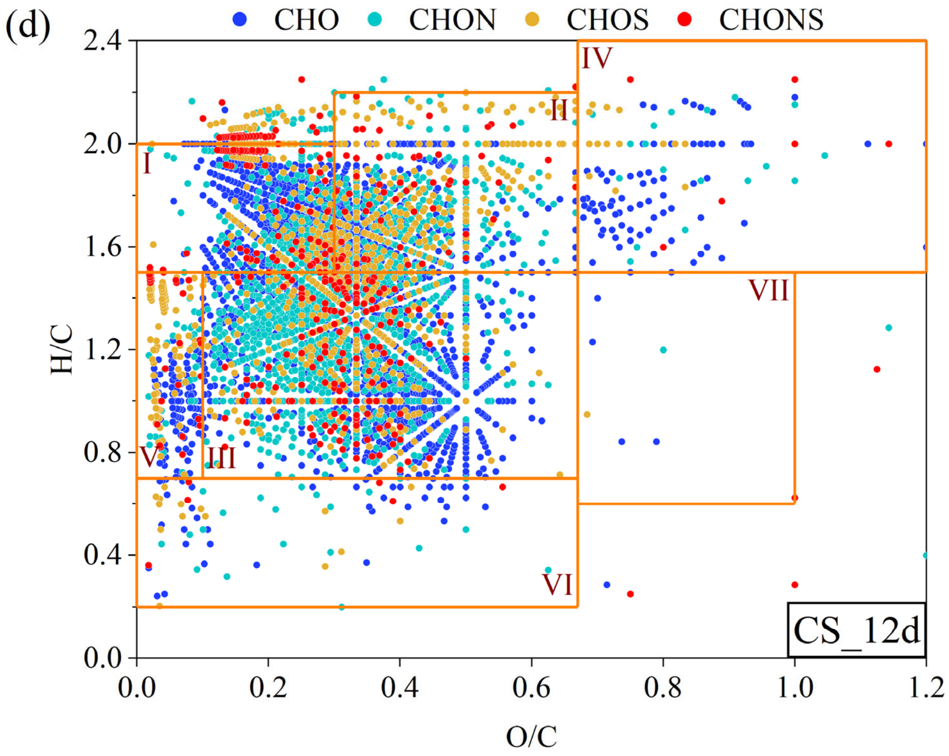
(d)
CHO
CHON
CHOS
CHONS
I
II
III
IV
V
VI
VII
CS_12d
H/C
O/C
0.0
0.4
0.8
1.2
1.6
2.0
2.4
0.2
0.6
1.0

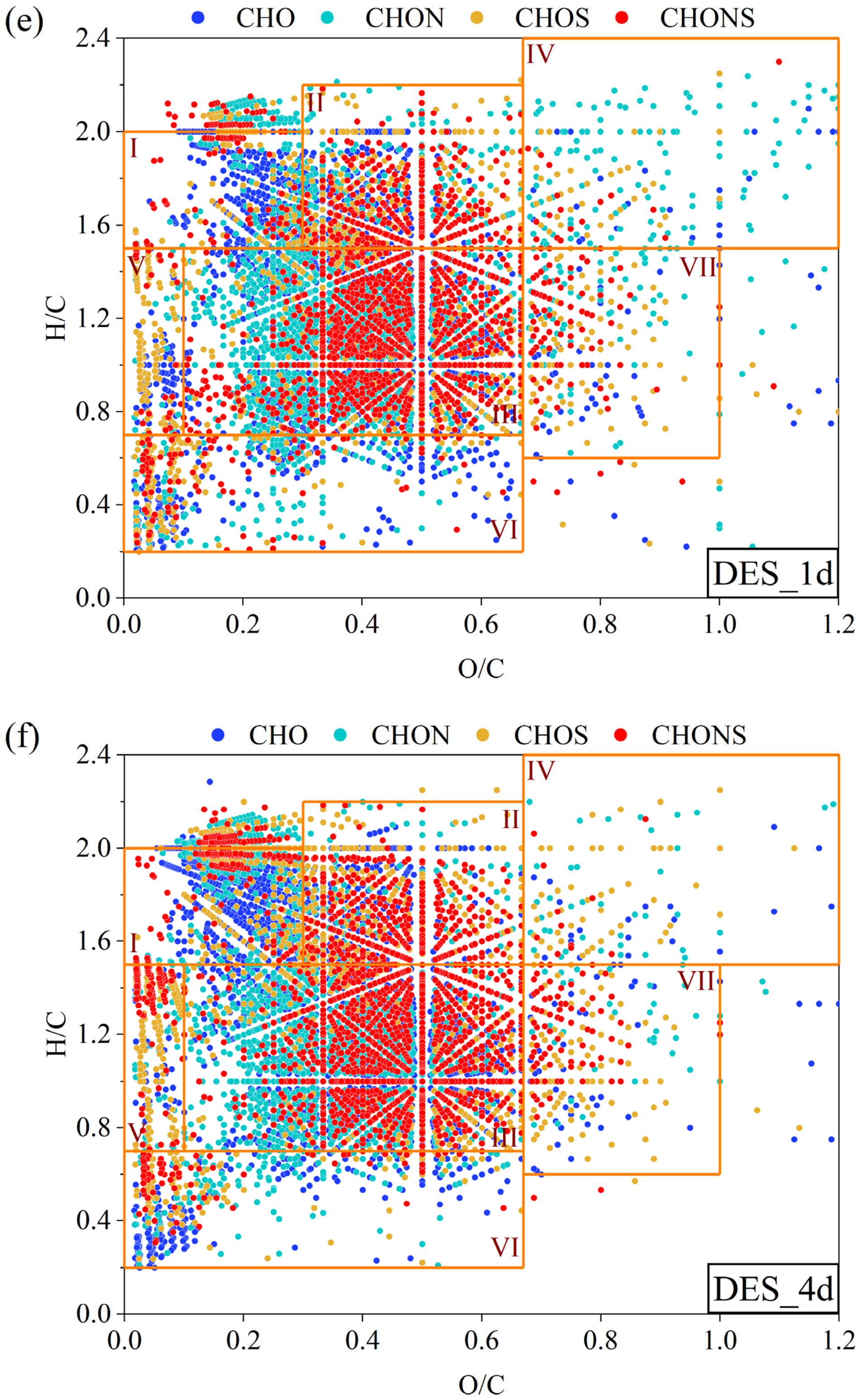
(e)
CHO
CHON
CHOS
CHONS
H/C
O/C
I
II
III
IV
V
VI
VII
DES_1d
(f)
CHO
CHON
CHOS
CHONS
H/C
O/C
I
II
III
IV
V
VI
VII
DES_4d

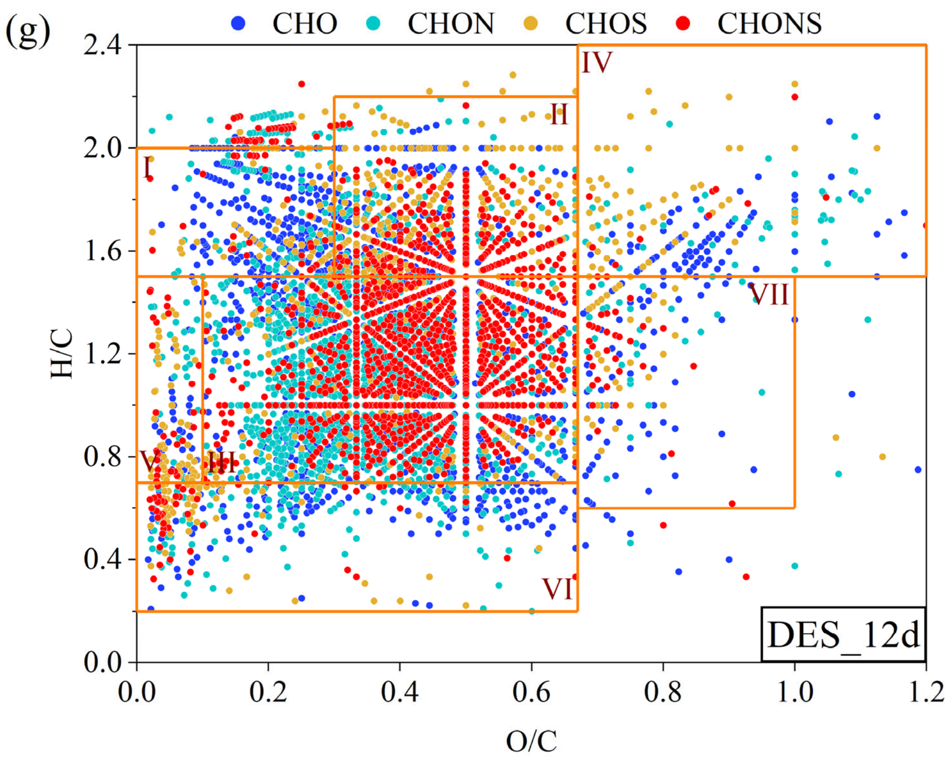

Fig S2. VK diagram of DOM components at different time of anaerobic digestion system of initial anaerobic granular sludge (a), CS (b, c, d) and DES group (e, f, g). AGS_0h represents the initial anaerobic granular sludge. CS_1d, CS_4d and CS_12d correspond to days 1, 4 and 12 of anaerobic digestion of untreated corn stover, respectively. DES_1d, DES_4d and DES_12d indicate samples collected on days 1, 4 and 12 during anaerobic digestion of DES-pretreated corn stover

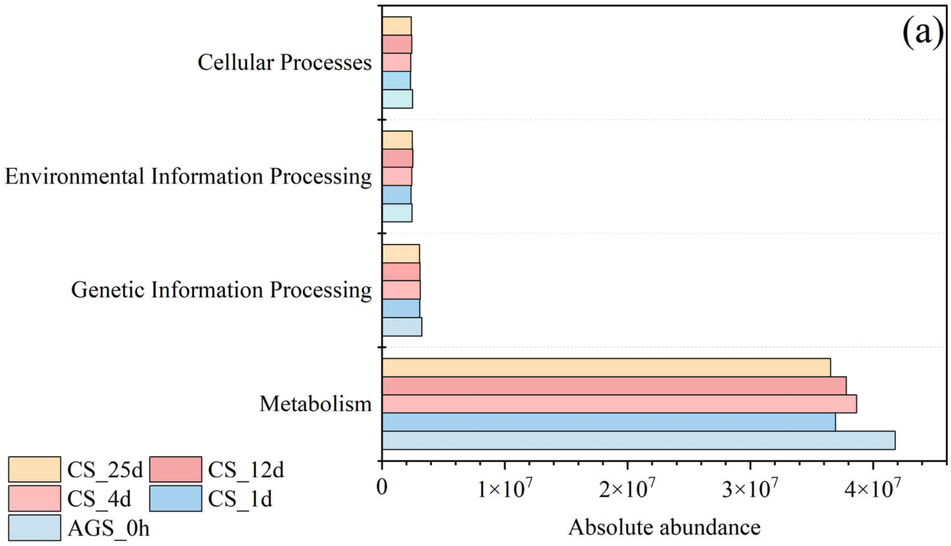

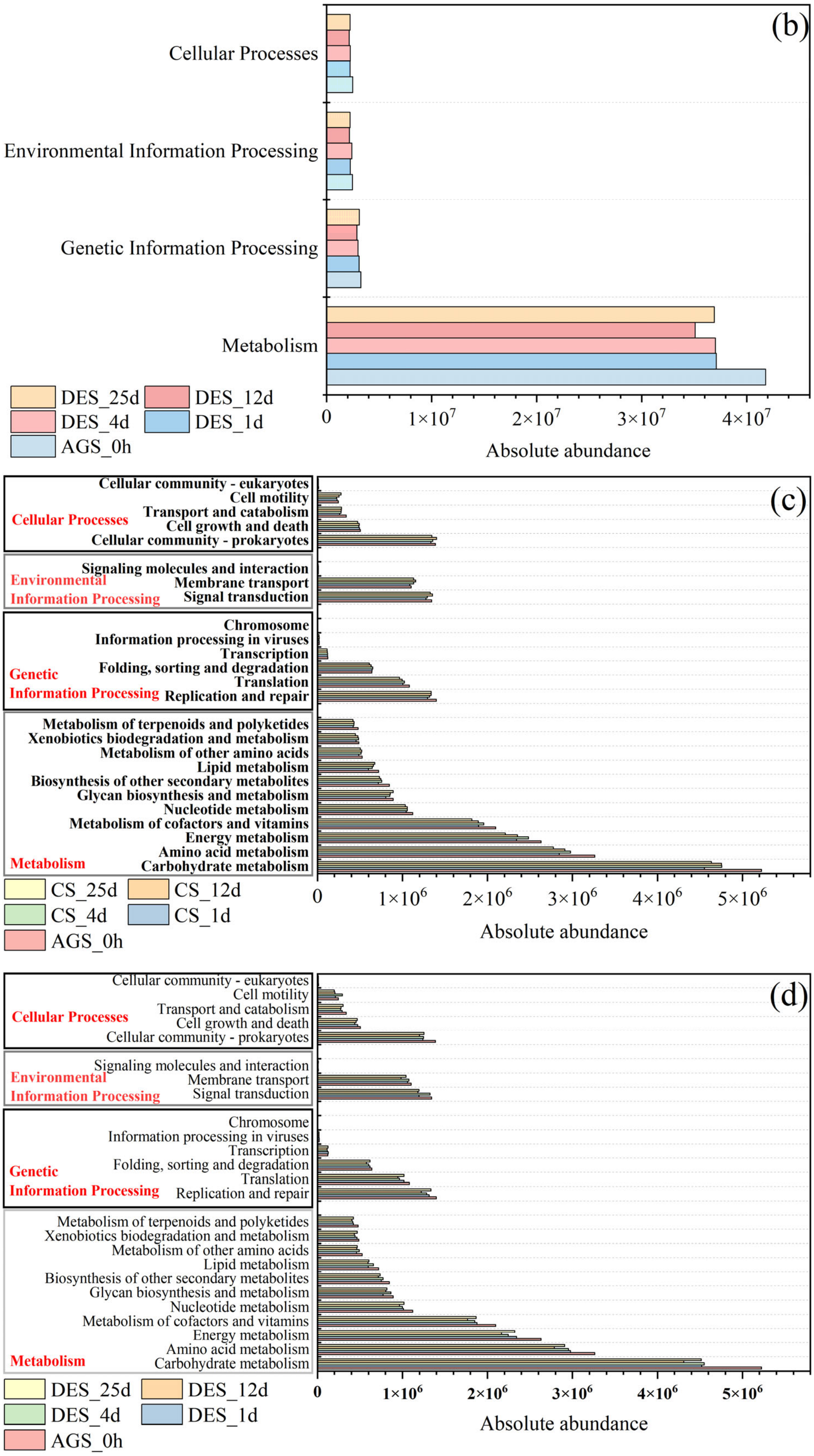

Fig S3. Absolute abundance of microbial functional pathways annotated at KEGG level 1 (a)(b), level 2 (c)(d) in CS and DES-pretreated corn stover AD groups

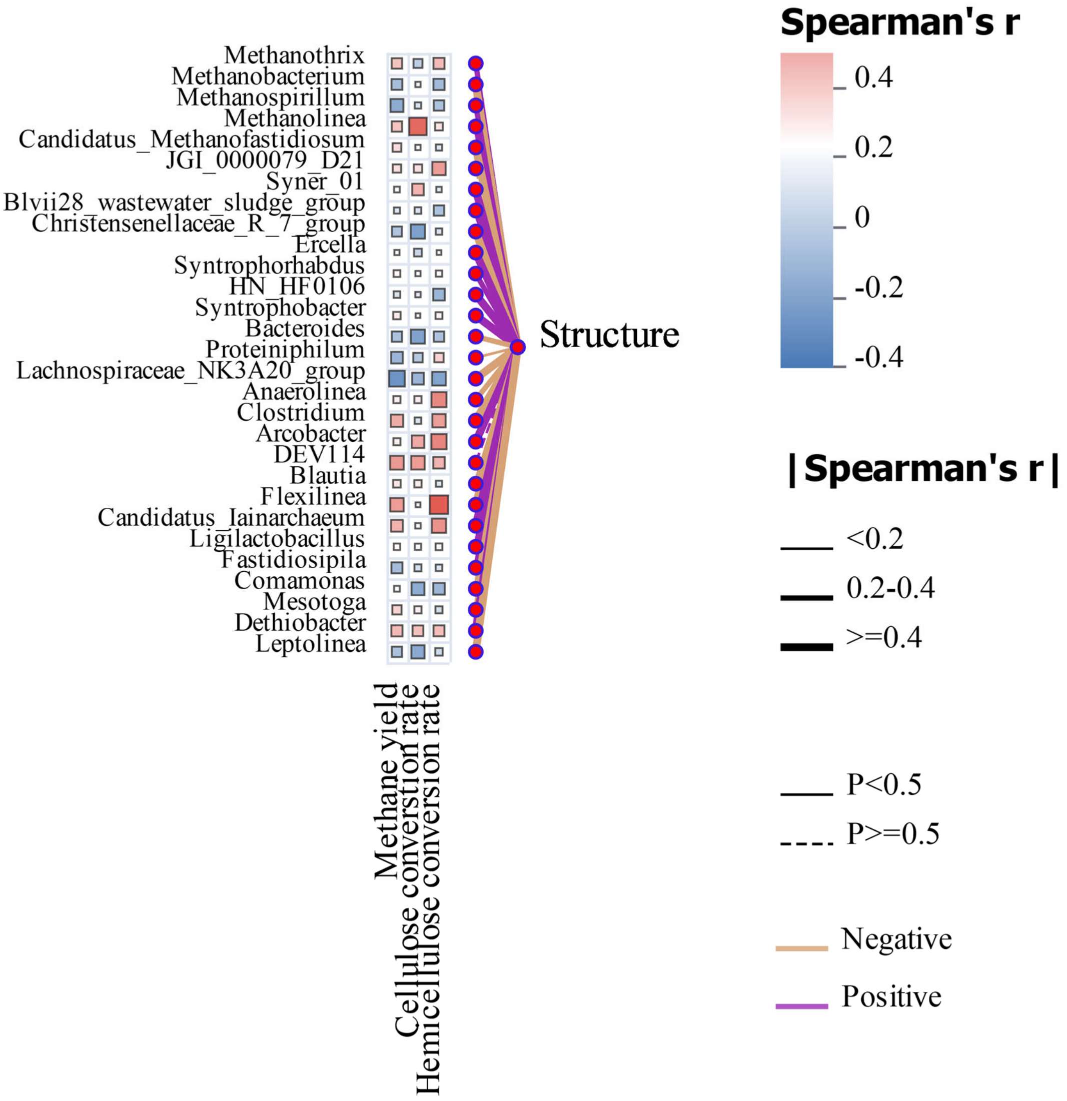

Fig S4. Correlation between microbial community composition and key fermentation performance indicators during anaerobic digestion. Spearman correlation analysis showing the associations between microbial genera and key anaerobic digestion performance indicators (methane yield, cellulose conversion rate, and hemicellulose conversion rate). The color of the lines indicates the direction of the correlation (purple: positive correlation; orange: negative correlation), and the line thickness represents the absolute value of the Spearman's correlation coefficient (|r|). Solid lines denote significant correlations ($P < 0.05$), while dashed lines indicate non-significant correlations ($P \geq 0.05$). The small squares on the left further illustrate the correlation coefficients (Spearman's r) between each microbial genus and individual performance indicators, with red representing positive correlations and blue representing negative correlations.Structure, defined as 0 for pure lignocellulosic individual components and 1 for corn stover.

Table S1. Molecular characterization of DOM in CK and DES-pretreated groups

Parameters	AGS_0h	CS_1d	CS_4d	CS_12d	DES_1d	DES_4d	DES_12d
CHO (%)	44.31%	42.28%	46.80%	54.46%	35.65%	40.62%	33.20%
CHON (%)	35.56%	19.63%	26.84%	26.36%	33.82%	28.61%	40.43%
CHOS (%)	9.03%	11.55%	18.03%	14.22%	16.64%	14.27%	14.51%
CHONS (%)	11.10%	26.54%	8.34%	4.96%	13.88%	16.50%	11.86%
Formula (n)	7844	5147	4275	6053	9941	10446	7936
Ave MS	387.4	480.31	422.78	427.27	419.31	468.1	439.33
H/Cwa（%）	1.34	1.63	1.57	1.49	1.33	1.3	1.33
O/Cwa（%）	0.36	0.25	0.31	0.31	0.36	0.41	0.36
DBEwa	7.79	6.09	6.48	7.03	9.17	9.22	9.17
AImod_wa	0.23	0.09	0.08	0.14	0.22	0.19	0.22
NSOCwa	-0.47	-1.03	-0.84	-0.78	-0.49	-0.33	-0.49
Lipids (I)	11.00%	29.99%	22.07%	20.73%	5.30%	14.71%	13.28%
Aliphatic/proteins (II)	22.97%	24.79%	32.28%	27.81%	18.55%	17.60%	20.55%
Lignin/CRAM_like (III)	62.57%	35.00%	32.93%	45.42%	56.14%	51.54%	49.60%
Carbohydrates (IV)	0.55%	0.87%	4.32%	2.27%	2.31%	0.91%	6.38%
Unsaturated hydrocarbon (V)	0.94%	7.59%	7.71%	3.07%	1.78%	8.25%	2.13%
Aromatic structures (VI)	1.75%	1.54%	0.65%	0.67%	11.68%	4.32%	5.88%
Tannins (VII)	0.22%	0.21%	0.04%	0.03%	4.23%	2.68%	2.18%

The values are abundance-weighted. Ave MS, average molecular mass; O/C and H/C, average elemental ratios; Almod, modified aromaticity index; DBE, double bond equivalent; NOSC, nominal oxidation state of carbon; wa, weighted average of intensity.

Table S2. Alpha diversity indices of microbial communities in different substrate groups during anaerobic digestion

Groups	chao1	faith_pd	simpson	observed_features	shannon_entropy
AGS_0h	528.89	54.33	0.96	528	5.81
MCLI	955.24	81.41	0.98	953	7.19
XYLI	908.97	70.51	0.98	906	6.93
MXL	873.14	67.77	0.98	871	7.15
MCXY	906.64	77.17	0.98	906	7.1
MC	801.41	68.52	0.96	799	6.66
XY	833.33	71.95	0.98	831	7.28
LI	823.36	63.29	0.98	821	6.96

CS	395.18	38.81	0.89	393	4.98
H_CS	633.7	46.37	0.94	628	6.17
HU_CS	524.53	41.49	0.88	524	5.09
BS_CS	417.96	33.29	0.94	415	5.66
CaO_CS	465.2	42.43	0.84	461	4.53
BC_CS	493.39	41.04	0.91	489	5.39
DES_CS	758.63	68.57	0.97	758	6.87

The Chao1 index estimates microbial community richness by predicting the total number of species in a sample based on the abundance of rare taxa, with higher values indicating greater species richness. Faith's PD (phylogenetic diversity) measures the total branch length of the phylogenetic tree represented in a community, reflecting its evolutionary breadth, where higher values correspond to greater phylogenetic diversity. The Simpson index assesses community evenness and dominance; values closer to 1 indicate higher diversity with more evenly distributed species, while lower values suggest dominance by a small number of taxa. Observed features represent the number of distinct microbial taxa directly detected in each sample, serving as a direct measure of community richness. The Shannon entropy index evaluates overall microbial diversity by accounting for both species richness and evenness, with higher values indicating a more diverse and evenly distributed community.
